# A Hybrid Rule- and Large Language Model–Based Embodied Voice Assistant (GRACE) for Cognitive Stimulation in Older Adults: Usability Study Assessing Technical Feasibility, Technology Acceptance, and Working Alliance

**DOI:** 10.2196/76489

**Published:** 2025-12-18

**Authors:** Rasita Vinay, Ekaterina Uetova, Nora Camilla Tommila, Nikola Biller-Andorno, Tobias Kowatsch

**Affiliations:** 1 Institute of Biomedical Ethics and History of Medicine Faculty of Medicine University of Zurich Zurich Switzerland; 2 Department of Management, Technology, and Economics ETH Zurich Zurich Switzerland; 3 School of Computer Science Technological University Dublin Dublin Ireland; 4 Institute for Implementation Science in Health Care Faculty of Medicine University of Zurich Zurich Switzerland; 5 School of Medicine University of St. Gallen St Gallen Switzerland

**Keywords:** voice assistant, dementia, older adults, human-computer interaction, embodied conversational agent, cognitive intervention, digital health interventions, large language model

## Abstract

**Background:**

The health and economic burden of dementia has led the World Health Organization to recognize it as a public health priority. Although there currently does not exist a cure for dementia, there are multiple interventions aimed at preventing the risk of dementia and improving the quality of life of people with dementia. Voice assistants (VAs), particularly those using large language models (LLMs), have emerged as promising tools to deliver these interventions to older adults due to their accessible and natural interface.

**Objective:**

This pilot study aimed to evaluate the technical feasibility (ie, functional performance and usability) and user acceptance of the embodied rule-based and LLM VA GRACE, as well as the perceived strength of the collaborative relationship or working alliance, between GRACE and healthy older adults during the delivery of cognitive stimulation interventions.

**Methods:**

A pilot study was conducted with 21 healthy German-speaking adults aged 60 years and older. Participants interacted with GRACE in a laboratory setting for 10-15 minutes. The interaction involved a structured cognitive stimulation session using rule-based and LLM components. Data were collected using pre- and postinteraction questionnaires and semistructured interviews. Quantitative analysis included descriptive statistics and Wilcoxon signed rank tests. Qualitative data were analyzed thematically.

**Results:**

Participants rated GRACE positively, with statistically significant scores above neutral (*P*<.001 for perceived ease of use, usefulness, enjoyment, and working alliance; *P*=.009 for perceived control; and *P*=.009 for intention to continue interacting). Thematic analysis revealed that GRACE was perceived as easy to understand and unambiguous, friendly, and supportive, with intervention components viewed as enjoyable and appropriately challenging. Areas for improvement included personalization, response delays, and voice quality.

**Conclusions:**

The results suggest that embodied rule-based and LLM VAs like GRACE are feasible and well-received tools for delivering cognitive interventions to older adults. Future iterations will incorporate feedback and extend testing to individuals at risk for dementia.

## Introduction

### Background

Dementia is a collection of symptoms severely affecting memory, thinking, and other cognitive skills in an individual’s daily life [1]. The most common form of dementia is Alzheimer disease [2], which was found to be the eighth leading cause of death worldwide [3]. In 2019, the estimated global societal cost of dementia was US $1.3 trillion, of which 50% of the costs were associated with informal caregiving [4]. The World Health Organization recognizes dementia as a public health priority through its global action plan [5], which hopes to minimize its health and economic burden worldwide. While this plan includes various action areas, such as dementia awareness, risk reduction, and treatment, digital assistive technologies should also be explored as prevention and management strategies.

Digital assistive technologies have emerged as valuable tools in supporting individuals living with dementia by enhancing independence, safety, and quality of life [6]. Designed to address challenges such as memory loss, confusion, and communication difficulties, these technologies support the autonomy of people with dementia while also alleviating the burden on caregivers [7]. Among the various forms of digital assistive technologies, voice-based digital assistive technologies, or voice assistants (VAs), offer distinct advantages due to an intuitive and hands-free conversational interface and low digital literacy requirement [8]*.* Unlike many other digital tools that require physical interaction or complex navigation, VAs rely on natural language processing, allowing users to engage through simple verbal commands. VAs can seamlessly integrate into daily routines, providing real-time reminders and scheduling medication [9-12] and even offering cognitive stimulation [5,13,14]*.*

When VAs were generally assessed, they reported high user satisfaction for lifestyle interventions [15]. Prior research has also shown that the embodiment of these conversational VAs can further improve user experience [16].

Despite significant advances, a notable research gap remains in the development of embodied VAs that move beyond task-based support to deliver structured, evidence-based therapeutic interventions specifically tailored to people with early-stage dementia. While existing embodied VAs have demonstrated potential in offering reminders, companionship, and daily routine support, few have integrated clinically validated therapies [17]*.* Agents that do provide such therapeutic content, such as Eva [13], are often highly scripted, relying on predefined dialogue flows and rule-based interactions that limit personalization, adaptability, and natural dynamic conversation. To address this research gap, GRACE integrates cognitive stimulation therapy (CST) [18] with a hybrid architecture that combines rule-based logic and large language models (LLMs) to support more flexible, personalized, and empathetic conversations.

In a previous study, we used an embodied VA prototype, GRACE, to test user acceptance and working alliance between the user and the technology in healthy younger adults (18-60 years) [19,20]. User acceptance was assessed with constructs of perceived ease of use (PEU), perceived usefulness (PU), and perceived enjoyment (PEN) [21-23]. Working alliance, which can be understood as the perceived strength of the collaborative relationship between GRACE and the user, similar to a doctor-patient relationship, was measured through the 6-item Session Alliance Inventory [24]. The user acceptance constructs were selected based on the research aims of this study and consistent with prior work on conversational agents in digital health [25-27], rather than to test a specific theoretical framework (eg, the technology acceptance model). Results of this first study were used to optimize GRACE (version 1), including its visual features, interaction script, language (English to German), and voice quality.

This paper presents the results of our second pilot study, which evaluated GRACE (version 2) with healthy older adults (60 years and older). To extend our earlier work, we added the constructs of perceived control (PC) and intention to continue interacting (ITI) [23,28], which have also been applied in related studies of conversational agents [25-27]. Technical feasibility, which we defined as the usability and functional performance of GRACE when used by healthy older adults, was evaluated through descriptive analysis of user acceptance and working alliance constructs, as well as qualitative feedback gathered from a postinteraction interview. This interview also evaluated the ethical dimensions observed within the technology by users, using values presented in the European Union’s Assessment List for Trustworthy Artificial Intelligence [29]. However, the qualitative findings from the interviews fall outside the scope of this paper and will be published in a separate paper that targets the bioethics research community.

### Related Work

#### Older Adults and VAs

A growing area of interest among researchers involves leveraging VAs to support the health and well-being of older adults [17,30,31]. VAs have the potential to support older adults’ independence by facilitating daily activities through smooth, speech-based communication, offering a user-friendly interface that requires minimal technological expertise and can accommodate varying levels of digital literacy [17,32-34]. Despite stereotypes about older adults’ reluctance toward technology, studies have shown that older adults often have positive attitudes and are open to using these devices in their daily lives [17,32,35-38]. It was found that older adults felt VAs might be useful for alerts of serious illness, health information management, and search, facilitating collaboration between themselves and caregivers [31,39,40]. At the same time, older adults were concerned about confidentiality risks and receiving trusted information [31,39] and skeptical about personal privacy and relational trust [40].

Numerous VA applications have been developed to support older adults, generally falling into 2 main categories: conversational social robots, which have a physical presence and use multiple affective communication modalities such as facial expressions, gestures, or body movements; and virtual artificial intelligence (AI) assistants or smart speakers, which primarily rely on voice interaction [17]. GRACE falls into the first category—a conversational social robot that engages users through verbal and nonverbal cues (facial expressions). A recent systematic review highlighted the perception of conversational AI among older adults, where they emphasized the need for empathetic, personalized, and context-aware interactions [41]. Current technologies in aging and early dementia care primarily aim to support individuals by promoting independence, enhancing social engagement, improving home safety, and monitoring health and well-being [42]. These applications can serve various roles; some are designed to act as companions for older adults experiencing loneliness [43]. For example, a “digital radio” system integrated voice-controlled features to enhance older adults’ connection with their family and address their needs for voice companionship, emotional interaction, and mental and physical well-being [44]. A companion robot “E-Bot” with integrated Amazon Alexa was designed to support older people living alone by enabling continuous interactions with caregivers, family members, and physicians while also monitoring physiological signals via wearable devices and an AI-enabled camera connected through Amazon AWS [45]. The NAO Robot provides single-session interactions, during which it reads newspapers to older adults, asks personal questions about their past, and listens to them, nodding occasionally and maintaining eye contact [46]. Studies have shown that robotic pets, which respond to user speech and petting with sounds and eye or body movements, can enhance communication, interaction skills, and activity participation of older adults [47].

VAs can also serve as home assistants, helping individuals manage their daily schedules. For instance, the Google Home Mini smart speaker can help cue individuals to take scheduled medications [9]. A companion bot named “Hello Steve” can send emails, open YouTube for entertainment, and track medication schedules to provide timely reminders for older users [10]. Another VA called “Mary” can assist older adults with daily tasks such as reminders, guidance with household activities, and locating objects [11]. Co-design approaches have also been explored with older adults to support health self-management, where the study found success in medication adherence and health-tracking features [12].

VAs have also been developed to provide therapeutic interventions for older adults. For example, VAs can be used for the prevention and management of chronic and mental health conditions [8], such as depression [48], encouraging physical activity [49], providing cognitive support [13,14], including VAs using LLMs with prompt engineering [50], and assisting with palliative care [51].

Despite this progress, most existing systems, including NAO, Hello Steve, and Mary, are limited in several key areas. First, these systems often rely on scripted interactions, which restrict their ability to provide personalized and context-aware engagement. Second, while some systems offer companionship or support daily living, few have integrated clinically validated interventions such as CST. Furthermore, the majority of VA applications are not co-designed with people living with dementia and their caregivers, resulting in limited adaptability to users’ changing needs and preferences over time.

To address these gaps, GRACE was designed as an embodied VA that moves beyond basic reminders and social dialogue to deliver structured, evidence-based cognitive interventions. By combining CST with hybrid rule-based and LLM-powered dialogue and by grounding development in stakeholder-driven design, GRACE aims to provide sustained, meaningful, and personalized support for individuals with early-stage dementia, advancing the field toward truly person-centered digital health technologies.

#### Rule-Based Versus LLM VAs

Most current VAs operate as passive, rule-based systems that respond only to wake words, requiring users to adapt their communication style to achieve desired outcomes [52,53]. While this simplicity can make interactions straightforward, it often leads to frustration when the system cannot respond to user requests [52,54]. LLMs present a significant advancement, enabling more adaptive interactions by better “understanding” human requests and overcoming the limitations of traditional, rule-based voice interfaces [52]. Nevertheless, both systems have their strengths and limitations.

Rule-based VAs offer advantages such as transparency, reproducibility, and safety, as their functionality relies on predefined rules, allowing developers to maintain full oversight due to their predictable responses [52]. However, rule-based systems are limited by their lack of flexibility and adaptability, as they cannot handle out-of-scope queries or adjust their personality and the challenges of scaling and maintaining complex multiturn interactions [52].

LLM VAs, on the other hand, overcome many of the challenges faced by rule-based VAs [52]. LLMs can process complex queries, retain context in multiturn dynamic conversations where user interactions are less predictable, and generate nuanced, more human-like responses [52,55]. However, these advantages come with drawbacks. LLMs’ reliance on large-scale computational resources raises challenges related to latency, particularly in real-time applications [56], hallucinations, that is, LLMs can generate nonsensical, incorrect text [57], unpredictable and unexpected responses [52], and bias [58,59].

#### Human Interactions With LLM VAs

LLM VAs have been shown to enhance individuals’ interactions to be more natural and effective. For example, FunAudioLLM models improve multilingual speech recognition and generation, enabling applications like emotional voice chat and speech-to-speech translation [60]. Additionally, Sesame AI showed the ability to reproduce natural pauses and intonation during conversations [61]. A study on VAs demonstrated that the integration of LLMs enhances conversational adaptability, enabling more nuanced and context-aware responses, while effectively addressing intent recognition failures and conversational breakdowns [62]. A study on LLM-powered robots showed that they excel in fostering connections and deliberation, but they struggle with logical communication and can sometimes cause anxiety [63]. However, more recent studies on LLM social-assistive robots found that older adults could successfully engage and improve on cognitive health-promoting tasks [64]. Another study introduced a framework for integrating LLMs as speech interfaces for assistive robots, demonstrated with the Obi feeding robot [65]. This highlights the importance of social capabilities that are often overlooked in physically assistive robots to enhance user engagement through social interaction. Talk2Care, an LLM asynchronous communication system designed to connect older adults at home with health care providers, demonstrated its potential to address communication challenges, enrich health information shared by older adults, and reduce providers’ workload [66].

In this and previous work [20], we address the limitations of traditional rule-based VAs while leveraging the strengths of LLMs by extending the rule-based language [67] with OpenAI’s application programming interface [68], enabling an embodied VA to combine the transparency and predictability of rule-based systems with the adaptability of LLM-generated responses within predefined, transparent boundaries.

## Methods

### Developing the Embodied GRACE Robot

**Figure 1:**
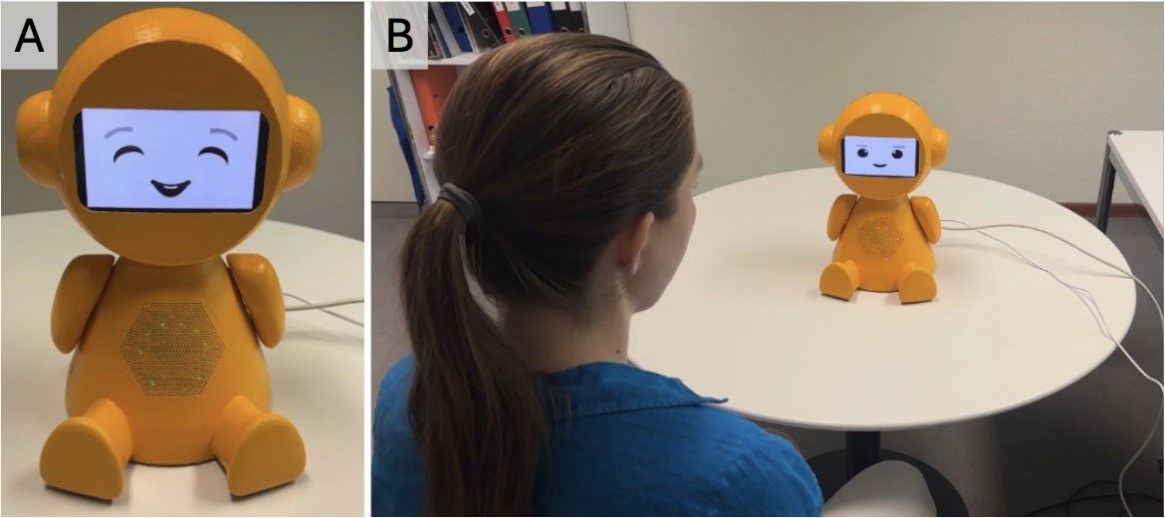
GRACE (version 2) prototype used for this pilot study: (A) front-facing image and (B) with a test user.

GRACE was 3D-printed with polyethylene terephthalate (a type of polyester plastic) using designs from an open-source social robotics platform [69], with its color changed from gray to bright yellow based on feedback from our previous study [20]. GRACE has a reSpeaker 4-Mic Array microphone, an LED ring, a 5.5-inch Waveshare active-matrix organic LED screen, an 8 GB Raspberry Pi 4 Model B, and a Xiaomi Bluetooth speaker. GRACE needs to be connected to power and a wired internet. Communication between the Raspberry Pi and an external computer uses the Message Queuing Telemetry Transport protocol. The external computer runs software [67] that executes interaction scripts or simulates them for development without GRACE’s body. GRACE’s modular Python-based software (Python Software Foundation) includes LED, audio, speech-to-text, text-to-speech, and display components [69]. The LED lights, green for listening to the individual and red for when GRACE is speaking or processing information, can be seen from the mesh in the body of GRACE (Figure 1). Audio output uses a Bluetooth speaker placed in GRACE’s head. The speech-to-text module uses Google Speech Recognition during <listen/> commands in the interaction script, while <talk> commands trigger the ElevenLabs service (Eleven Multilingual version 2) [70] to generate natural-sounding German speech (“Leonie” voice), cached for reuse. The screen shows animated facial expressions like blinking and smiling.

### Design of Conversational Turns

The interaction script, adapted from a pilot study [20], was first drafted in plain text, and then, converted to XML with commands like <talk> and <listen/> (the full interaction script can be found in Multimedia Appendix 1) [67]. GRACE responds with predefined answers or dynamic replies via OpenAI’s application programming interface [68]. This hybrid approach allows us to maintain consistency and control of rule-based interactions while enabling more nuanced and context-aware conversations, addressing the challenges faced by traditional systems and LLM VAs.

The script is based on CST and includes 3 intervention components and 2 additional activities (onboarding and conclusion). CST is an evidence-based group therapy program for people with dementia, encompassing a variety of themed activities (ie, word games, reminiscence, and creative games) to improve well-being and cognition [71,72]. A CST manual was used to guide development of the GRACE sessions as well as to identify different activities that could be included as part of the intervention components [73]. The script starts with an onboarding activity (supported by LLM), where GRACE introduces itself to the participant and engages in conversation to get to know the participants (ie, by asking questions such as “Do you have siblings?”, “Do you have a favorite hobby?”, and “What is your hometown?”). In this interaction, the responses from GRACE are LLM-generated, where we prompted the LLM to provide a supportive statement or fun fact based on the participants’ answer (see Multimedia Appendix 2 for the LLM prompts used in the complete interaction). After this, GRACE provides participants with an overview of the interventions it will offer.

The first intervention is a warm-up activity in the form of a guessing game, where different animal sounds are played, and the participant must guess the name of the animal that makes that sound. This interaction is rule-based with stored audio files playing selected animal sounds (ie, a cow, cat, dog, rooster, and horse), and GRACE responding with the name of the correct animal. Next, the participant can choose which intervention they want to complete first, a memory or breathing exercise. By allowing participants to select the order of the intervention, our goal is to promote autonomy and motivation, in line with the self-determination theory [74]. In the memory exercise, GRACE reads a short shopping list (1-5 items, an LLM is used to help determine the individual’s answers and correct item recall), and the participant is asked to repeat the list in any order. If the participant cannot list all the items, GRACE repeats the missed items. The breathing exercise is rule-based and consists of a short, guided breathing training based on exhale-focused cyclic sighing [75]. GRACE allows participants to select from 3 acoustic relaxing environments (stored audio files of the forest, the sea, and a cabin with a fireplace), which are used to immerse them in the training environment through background noises and a description of the environment by GRACE. There is no participant communication beyond the environment selection; the stored audio files guide users to follow inhale or exhale techniques for 2 minutes. In the rule-based concluding activity, GRACE thanks the participant for doing the exercises, asks them about their favorite activity, provides health education on the importance of completing cognitive interventions, and farewells with a reminder to stay active.

### Participants

Overall, 21 participants meeting the inclusion criteria took part in the study during August and November 2024. Study recruitment took place via email and posters, highlighting the study’s aims and the inclusion and exclusion criteria.

The inclusion criteria were as follows: aged 60 years and older, ability to speak and understand German (at a B1 level), and no cognitive or hearing impairments. The exclusion criteria were as follows: having severe psychiatric disorders or a history of substance abuse.

The study was conducted in a laboratory setting in a single sitting, with participation lasting approximately 45 minutes.

### Data Collection

The data collection consisted of a pre- and postinteraction questionnaire (Multimedia Appendices 3 and 4) and a short semistructured interview (Multimedia Appendix 5). The preinteraction questionnaire collected the participants’ age, gender demographics, and general information regarding experience with VAs.

The postinteraction questionnaire consisted of 12 items used to assess technology acceptance [21]. The 7-point Likert scales were used for each item and anchored from 1=strongly disagree to 7=strongly agree. Consistent with prior work on conversational agents [27], we used self-report instruments for PEU, PU, PEN, PC of GRACE, and the ITI with GRACE [21,28,76]. Working alliance, which is an important relationship quality and robustly linked to treatment outcomes [77-79], was measured with the 6-item Session Alliance Inventory [80].

A semistructured interview with 10 questions was conducted to collect qualitative feedback on the interaction with GRACE. Questions 1-7 were developed by authors RV and NB-A, based on the Assessment List for Trustworthy Artificial Intelligence checklist [29], on the principles of human agency and oversight, technical robustness and safety, privacy and data governance, transparency, diversity, nondiscrimination and fairness, societal and environmental well-being, and accountability. The results of interview questions 1-7 will be reported in a separate publication targeting the bioethics community. Questions 8-10 relate to the overall positive and negative aspects of interaction with GRACE and any further comments or suggestions that are reported in this work.

### Procedure

Participants completed the preinteraction questionnaire (approximately 1 minute) after providing informed consent. Participants were then given instructions for the interaction with GRACE (ie, what the different LED-light colors mean and speaking clearly, etc). Once the participants were ready, the interaction was started by a researcher, after which GRACE led the entire interaction independently, with supervision from the researcher. The interaction with GRACE lasted 10-15 minutes and followed the interaction script as mentioned earlier. Following the interaction, participants completed a postinteraction questionnaire (5 minutes) and were then interviewed by a researcher (15 minutes).

### Data Analysis

To summarize participants’ responses on the postinteraction questionnaire, means and SDs were computed for each quantitative self-reported item. As the Shapiro-Wilk tests revealed deviations from normality in the data distribution, nonparametric analysis was warranted. Therefore, Wilcoxon signed rank tests were applied to examine whether the median scores significantly diverged from the neutral midpoint value of 4 on the 7-point Likert scale. Additionally, a composite score for the 6-item Session Alliance Inventory was created by averaging responses across all items, supported by high internal consistency (Cronbach α=0.92) [81]. Consistent with prior work on embodied VAs [20,82], thematic maps were used to present the results of interview questions 8-10. Authors RV and NCT conducted 3 rounds of thematic analysis until a consensus was reached.

### Ethical Considerations

The study was exempted from formal review by the ethics committee of the University of St. Gallen on May 17, 2024. During the onboarding, participants were informed about the study’s aims, procedure, and rights as participants. They were then provided with a written consent form for participation. All participants were assigned a participant code to maintain anonymity of responses (indicated as P#, where # was a randomly assigned number from 01 to 21). No compensation was provided to the participants.

## Results

In total, 21 individuals (8 male and 13 female) participated in the study, where the average participant age was 69.3 (SD 6.2) years. From the preinteraction questionnaire, 8 participants indicated that they had used a VA previously, on average, a few times a month. Participants also indicated that they would use a VA if it provided them with activities and exercises to improve their quality of life (mean 5.38, SD 1.07, “somewhat agree” on the 7-point Likert scale).

Table 1 presents a summary of the quantitative evaluation (ie, technology acceptance and working alliance) of GRACE, showing that all mean scores exceeded the neutral midpoint of 4 on the scale. The postinteraction questionnaire revealed strong indicators of technological acceptance and a robust sense of working alliance with GRACE (mean 5.49, SD 0.81; Table 1). Participants rated the system as highly easy to use (mean 6.19, SD 0.81), suggesting that GRACE’s conversational interface and structure were well understood and accessible. Moreover, PEU, PU, PEN, and overall assessment of the working alliance had a significance of *P*<.001. PC had a significance of *P*=.009, ITI with GRACE had a significance of *P*=.009, and item 5 from the Session Alliance Inventory had a significance of *P=*.01. Therefore, it can be concluded that participants responded positively to GRACE.

**Table 1:** Postinteraction quantitative evaluation of 21 participants on their perceived ease of use (PEU), perceived usefulness (PU), perceived enjoyment (PEN), perceived control (PC), intention to continue interacting (ITI), and perceived session alliance (PSA)^a^.

	Item	Mean (SD)	*V* ^b^
PEU	I thought the response from GRACE was easy to understand.	6.19 (0.81)	231.0^c^
PU1	I found it useful to work on the intervention with GRACE.	5.76 (1.14)	205.5^c^
PU2	I found GRACE motivated me to perform the exercises.	5.48 (1.21)	178.5^c^
PEN	I had fun using GRACE.	5.95 (0.86)	231.0^c^
PC	I was able to control the interaction with GRACE.	5.19 (1.69)	174.0^d^
ITI	I would continue interacting with GRACE.	5.14 (1.53)	105.0^d^
PSA1	I felt that GRACE and I respected each other.	5.81 (1.25)	204.0^c^
PSA2	I felt that GRACE appreciated me.	5.57 (1.21)	198.0^c^
PSA3	I felt that GRACE cared about me even when I did things it did not approve of.	5.33 (1.06)	120.0^c^
PSA4	I felt that GRACE and I are working toward mutually agreed upon goals.	5.67 (1.15)	222.0^c^
PSA5	I felt that GRACE and I agree on what is important for me to work on.	5.10 (1.73)	121.0^e^
PSA6	I believe the way GRACE and I are working with my problem is correct.	5.48 (1.03)	136.0^c^
PSA	Overall	5.49 (1.06)	222.0^c^

^a^The 7-point Likert scales were used from 1=strongly disagree to 7=strongly agree, and a 6-point scale for PSA items.

^b^*V* is the test statistic of the Wilcoxon signed rank with test value 4.

^c^*P*<.001.

^d^*P*=.009.

^e^*P*=.01.

**Figure 2:**
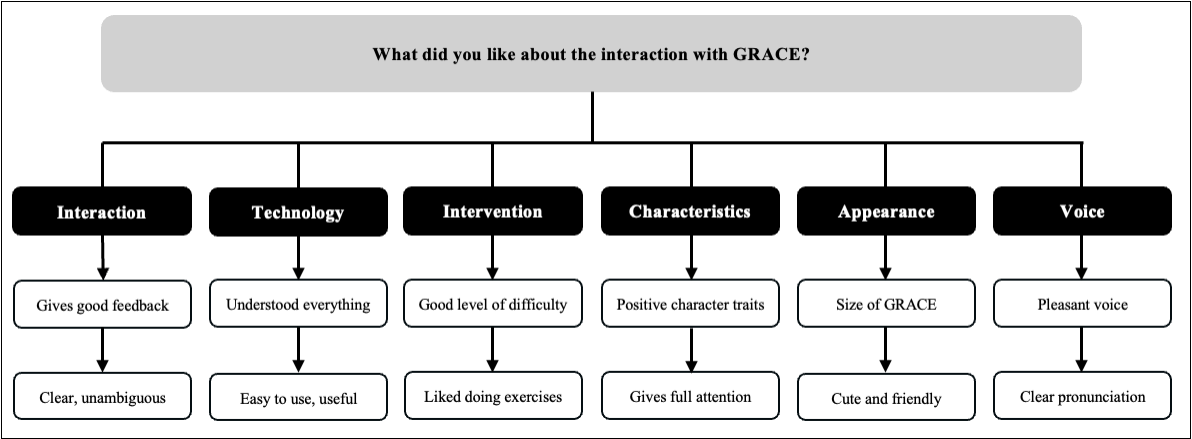
Thematic map representing answers of study participants regarding the question: “What did you like about the interaction with GRACE?”. Boxes in black indicate identified categories, and boxes in white indicate participant responses.

**Figure 3:**
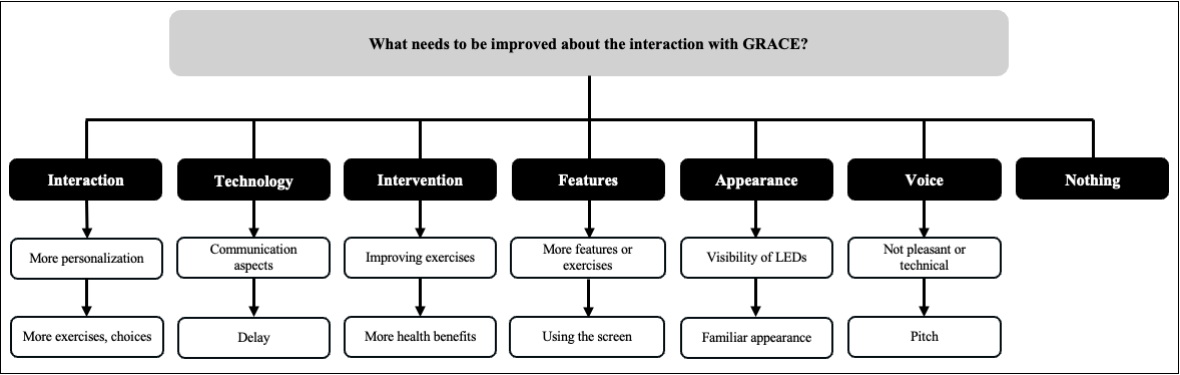
A thematic map representing answers of study participants regarding the question: “What needs to be improved about GRACE?”. Boxes in black indicate identified categories, and boxes in white indicate participant responses.

**Figure 4:**
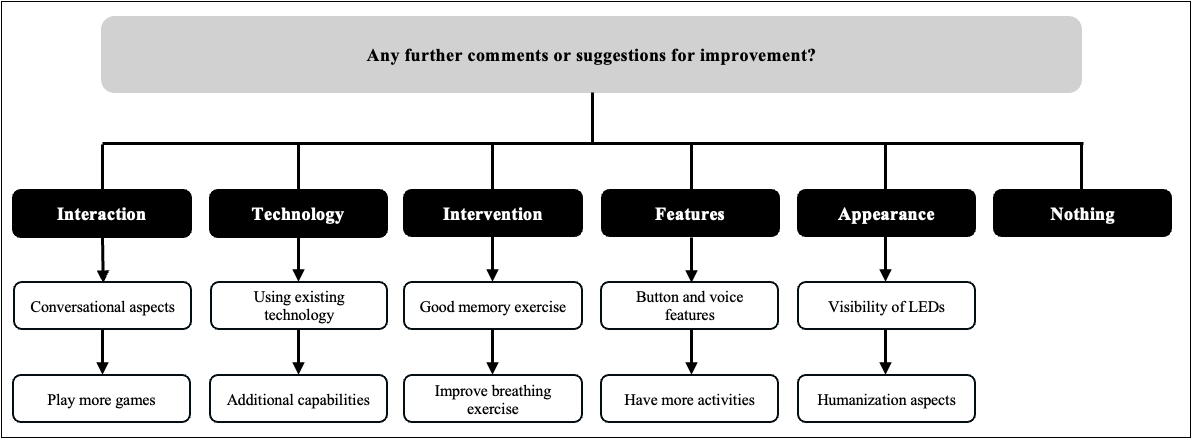
A thematic map representing answers of study participants regarding the question: “Any further comments or suggestions for improvement?”. Boxes in black indicate identified categories, and boxes in white indicate participant responses.

Following their interaction with GRACE, a semistructured interview was conducted with all participants. Thematic analysis of the responses revealed key insights into what participants appreciated (Figure 2), areas for improvement (Figure 3), and further comments or suggestions (Figure 4). Direct participant quotes for the 3 interview questions are included in Multimedia Appendix 6.

Participants expressed a wide range of positive impressions regarding their interaction with GRACE. This included the ease of understanding and using the system, the friendly and supportive nature of GRACE, and the usefulness of the exercises. GRACE was described as “clear and unambiguous” (P01), “pleasant and friendly” (P08), and “able to give feedback” (P05 and P11). Many appreciated the ability to interact at their own pace and felt that GRACE responded accurately and attentively, even to mumbled speech (P13 and P14). The intervention itself, such as the memory and breathing exercises, was seen as engaging and appropriately challenging (P05, P11, and P19) and not too stressful (P04, P08, and P15). Participants also responded positively to GRACE’s appearance and voice, describing her as “cute” and with “clear pronunciation” and a “pleasant voice” (P03, P08, P16, P19, and P21). Several participants also appreciated GRACE’s personality traits, perceiving her as “sympathetic” (P02), “polite” (P04), and able to offer “full attention” during the interaction (P06 and P12).

Despite overall satisfaction, participants identified several areas where GRACE could be improved, focusing on technology, interaction design, intervention content, and user experience. Technological aspects such as communication delays and accuracy were frequently mentioned: “Her reaction is a bit slow” (P20), “It didn’t always understand what I said” (P07), and “the communication is not great yet” (P21). The voice was another concern, described by some as “too technical” (P01), “not so pleasant” (P07), or “disturbing” due to pitch (P05). Participants desired more personalization and variety in interaction: “the answers were very stereotypical” (P08), and that “the exercises could be personalized” (P11 and P13). Some also found the content demanding or insufficiently explain: “the tasks and exercises were quite demanding” (P02) and “maybe it could explain the effects more, why should I do these exercises?” (P03). Many also felt the system to be “mechanical” (P20) and “impersonal” (P11), which prevented its ability to establish a more human connection (P12).

A range of additional reflections and suggestions to enhance GRACE’s functionality and relevance were also offered by participants in the last interview question. Some proposed expanding the system’s interactive capabilities, such as incorporating games (P06), adding a physical button (P08), or enabling more open-ended conversations (P12). Several comments emphasized the value of personalization, including the ability to choose a familiar image or voice: “Human face would be good or to be able to choose a photo, like of my grandson” (P08) and “maybe give also a choice for a nice, male voice” (P13). Others suggested improvements to specific features, such as enhancing the lighting (P07), adding more background sounds to the breathing exercise (P04), or clarifying the system’s nonhuman nature if a more lifelike appearance is used (P12). A few comments reflected on the context and potential of GRACE, including its suitability for people with dementia: “My mother responded to ‘garden’, ‘dog’, ‘favorite food’, ‘music’...” (P20) and how using personally meaningful cues could enhance engagement with GRACE. Others appreciated the potential for GRACE to support those who are lonely or in need of a companion-like presence: “It could be helpful to have an entity that asks questions and listens, even though it’s not a human interaction” (P12). The overarching tone of responses conveyed both interest and constructive engagement with GRACE’s future development.

## Discussion

### Principal Findings

This study aimed to assess technical feasibility, technology acceptance, and working alliance with GRACE in healthy older adults (60 years and older). The sample included a balanced group in terms of gender, with an average age of approximately 69 (SD 6.2) years, reflecting a relevant demographic for future health-related applications of VAs. While only a portion of participants reported prior experience with such technologies, there was a clear openness to engaging with GRACE, particularly when the system offered meaningful intervention components aimed at improving quality of life. Postinteraction feedback further supported this readiness: participants not only found GRACE highly usable and easy to navigate but also indicated a strong sense of working alliance with the system. This aligns with prior research, showing that older adults are receptive to voice interfaces when designed with clarity and intuitiveness in mind [83,84].

PU was also rated positively, with participants expressing that working with GRACE was beneficial (PU1: mean 5.76, SD 1.14) and motivating (PU2: mean 5.48, SD 1.21). Notably, PEN (mean 5.95, SD 0.86) was among the highest-rated constructs, indicating that beyond usability, participants genuinely enjoyed the interaction. These ratings support the potential for voice-based interventions to engage users in an emotionally positive way, which is particularly valuable for health-promoting interventions in older populations [8].

Participants also reported a relatively high sense of PC over the interaction (mean 5.19, SD 1.69). However, this dimension showed more variability, potentially reflecting differences in individual familiarity with conversational technologies or preferences for interaction pace. Intention to interact (mean 5.14, SD 1.53) suggests a moderate to strong willingness to engage with GRACE in the future, reinforcing the system’s potential for repeated use and longer-term integration into daily routines.

The assessment of perceived session alliance (PSA) further underscores the strength of the interaction. All 6 session alliance items scored well above the midpoint on a 6-point scale, with particularly high ratings for mutual respect (mean 5.81, SD 1.25) and feeling appreciated (mean 5.57, SD 1.21). These findings suggest that users not only felt heard and acknowledged, but also that GRACE was perceived as working collaboratively toward shared goals (eg, PSA-4: mean 5.67, SD 1.15). Although item PSA-5 (“I felt that GRACE and I agree on what is important for me to work on.”) received the lowest PSA rating (mean 5.10, SD 1.73), it remained significantly above the neutral test value, indicating room for improvement in tailoring goals and content to individual users.

In line with the quantitative findings, qualitative feedback highlighted positive perceptions of the interaction with GRACE. Participants described the interaction as “clear and unambiguous,” “well explained,” and “stress-free,” emphasizing the ease of use and the feeling of being understood. Many found GRACE’s voice and appearance to be “pleasant,” “cute,” or “friendly,” with others noting she gave “good feedback” and created a sense of receiving full attention, even if it was “pseudo-interest.” Several participants appreciated the exercises, especially memory and breathing exercises, citing them as engaging and motivating. GRACE’s character was also described as “sympathetic,” “positive,” and “polite,” contributing to the experience of a supportive and approachable interaction partner.

At the same time, participants offered constructive suggestions for improvement, pointing to areas that could strengthen engagement and usability. Most notably, concerns were raised around the technical quality of the voice (eg, “too technical,” “not so pleasant,” and “mechanical”), slow system responses, and occasional misunderstandings in speech recognition. Some participants desired greater personalization in the interaction, such as tailoring the pace of exercises or receiving feedback that felt less generic. Suggestions also included expanding the scope of features, such as adding a button, screen use, or more varied exercises, and incorporating a more human or familiar appearance, while carefully clarifying that GRACE is not a real person. A few noted that the interaction felt “short” or “superficial,” suggesting potential for deeper engagement in relational continuity in future iterations.

Additional comments reiterated the need for more flexibility, both in terms of content and delivery. Participants proposed more background noises for the breathing exercise, more open conversational exchanges, and the option to personalize GRACE’s appearance or voice (eg, offering a male voice or selecting a familiar face). While some questioned the added value over existing VAs like Siri, others particularly emphasized GRACE’s potential in supporting people experiencing loneliness or cognitive decline. A particularly insightful remark highlighted that “GRACE’s productivity is not just about reducing loneliness” but also about sparking memory, interest, and emotional connection through personalized keywords or emotionally salient cues, which may be relevant for future use in dementia care.

Altogether, these results provide compelling early evidence that GRACE can foster both functional and relational engagement with older adults, meeting core criteria of usability, enjoyment, and alliance formation. When interpreted alongside the qualitative feedback, it becomes clear that the perceived success of GRACE hinges not only on task performance but also on emotional, aesthetic, and interpersonal dimensions that contribute to a meaningful user experience.

### Comparison With Own Prior Work

This section briefly compares the significant improvement of outcomes in this study with a previous pilot study that was conducted with an earlier version of GRACE in healthy younger adults [19,20]. Here, several key differences can be highlighted.

#### Design Enhancement

In response to prior feedback, GRACE’s color was adjusted to create a warmer and more inviting visual appearance (from gray to yellow/orange). Additionally, an LED light was also inserted to indicate to users when GRACE was waiting for a response and when it was not. These design shifts may have positively influenced user perception and emotional engagement with the system. Furthermore, the current version of GRACE delivered an additional intervention—an animal guessing game, which may help users become more familiar with its interaction style earlier on. The idea behind this additional intervention was to help users get familiar with interacting with GRACE in an exercise and add more cognitive interventions as part of GRACE’s service. Therefore, adding this simple intervention could potentially reduce initial resistance and confusion to interaction and improve receptivity to the system’s guidance.

#### Better Alignment With the Target Population

Participants in this pilot study were more closely matched with the intended user group (people with early dementia) regarding demographics and needs. The closer alignment likely contributed to increased relevance, engagement, and PU of the system. The idea behind slowly moving toward the target population was to ensure a safe and ethical approach to reaching vulnerable population groups, where the technology should first be tested among healthy adults, reiterated, tested among healthy older adults, reiterated, and then finally presented to other relevant stakeholders involved in dementia care. Once more, feedback is incorporated from this group of stakeholders; only then would GRACE be tested among people with early dementia.

#### Use of Participants’ Native Language

Unlike the first pilot study, this study conducted interactions in the participants’ native language—German. Language is a crucial component for trust building and user comprehension. Conducting the interaction in high German (not Swiss German) likely reduced the participants’ cognitive load, minimized misunderstandings, and even made the system feel more inclusive and accessible. In multilingual settings, such as those in Switzerland where the study was conducted, even subtle linguistic mismatches can impact the perception of relevance and credibility. By aligning linguistic communication with participants’ cultural context, this study likely enabled more natural interactions and a deeper sense of being understood. Moreover, native language may have helped convey empathy and nuance, which are particularly important in health and well-being domains, where the tone and phrasing matter greatly.

### Comparison With Related Work

Our findings align with previous research, indicating that older adults are receptive to VAs, particularly when these are designed to be intuitive, emotionally engaging, and relevant to users’ needs [17,32,35-38]. GRACE’s high usability and enjoyment ratings echo studies emphasizing the value of speech-based, low-barrier interfaces for older populations [33,34]. Similar to social robots like NAO or robotic pets [46,47], GRACE fostered a sense of being heard and respected, supporting past evidence that effective engagement enhances users’ experience.

A recent study has also started exploring the use of VAs to deliver individual CST to people with dementia, initially tested with a very small sample of family members and informal caregivers [85]. Their preliminary findings hinted at acceptance and technical feasibility by the participants. Based on this pilot study, we believe that GRACE provides a more holistic approach to CST by embedding engaging socially interactive intervention components to enhance emotional connection and provide cognitive support and user engagement.

GRACE’s hybrid rule-based and LLM design may explain the balance between PC and conversational flexibility. This reflects recent work, suggesting that combining transparency with adaptability can improve trust and usability in health-focused VAs [52,62,65]. However, some participants still perceive GRACE’s responses as “mechanical,” consistent with concerns about emotional nuance and speech naturalness in LLM systems [57,63]. This echoes broader critiques of LLMs in the health care context, particularly regarding their ability to generate responses that are grammatically correct but factually inaccurate, emotionally misaligned, or culturally insensitive, which poses particular risks when interacting with older adults or individuals with cognitive impairments [86]. Moreover, the opacity of LLM-generated dialogue poses ethical challenges around accountability and safety, especially when users interpret the VA’s output as clinically authoritative [87]. Additionally, the opaque nature of LLM decision-making makes it difficult for users to anticipate or control responses, which may undermine trust [88]*.* Requests for personalization (eg, voice, pace, and feedback content) resonate with studies calling for more tailored and culturally sensitive VA designs in older adult care [10,12,39,66]. Notably, some users experienced GRACE as more than just a tool, highlighting memory, meaning, and emotional connection, which extend prior findings on the potential of VAs to support not only functionality but also relational and emotional well-being [40,44,51].

Our findings underscore the importance of control and personalization in the design of VAs for older adults and dementia care. Prior studies have shown that older adults emphasize the importance of thoughtful language, appropriate tone, and personalization when designing conversational agents for individuals affected by dementia [89]. Moreover, older adults value the ability to control the interaction and engage with systems that reflect cultural and contextual relevance [90,91]. Cultural appropriateness, language use, and visual or conversational representation play a key role in making intelligent systems feel relatable and comfortable to use, particularly among racially and culturally diverse older adults [92]*.* Furthermore, users’ perceptions of empathy are shaped by how well the systems acknowledge and respond to aspects of users’ identity, such as race, disability, and lived health care experiences [93].

### Limitations and Future Work

In future work, we plan to reiterate GRACE based on this study’s feedback and conduct interviews with other stakeholders such as caregivers, family members, and health care professionals to obtain their perspectives on GRACE. Such insights could be beneficial in identifying other interventions that may be useful for people with dementia or ways in which communication between GRACE and the user can be enhanced. It may also be beneficial to speak to health insurers and service providers to identify scalable solutions for implementing GRACE within the dementia community, so that they may be considered for future research directions that focus more on the implementation of GRACE within the community.

Although this study shows preliminary results that delivering interventions to older adults with embodied VAs is positively perceived, the effects of the interventions and the relationship between them remain unknown. A next step would be to test such interventions with individuals at risk of developing dementia, that is, those with mild cognitive impairments or subjective cognitive decline, to get closer to the target population. Additionally, integration of interventions, such as reminiscence therapy, which allows older adults to engage in conversations that evoke meaningful memories and foster emotional well-being [94], or cognitive storytelling [95,96], has shown promise in improving mood, reducing stress, and enhancing cognitive engagement in older adults [94-96]. These intervention examples may be used in future versions of GRACE, arising from existing research.

A potential limitation of this study is the possibility of social desirability bias, whereby participants may have provided more positive evaluations than they felt [97]. This bias can be particularly relevant in face-to-face settings, where participants may wish to please the research team or provide favorable evaluations of a new technology. While we cannot fully exclude this bias in participants’ responses, we actively mitigated the risks by using anonymized questionnaires and having the interviews conducted by student researchers outside the study team. These measures reduce the likelihood that participants felt pressured in their responses, and that the consistently high ratings likely reflect the system’s design refinements and closer alignment with the target population. Nonetheless, subtle forms of social desirability bias may still have influenced the findings, as is common in studies relying on self-reported perceptions.

Another potential limitation that must be mentioned is that the postinteraction technology acceptance questionnaire was constructed ad hoc from items adapted from prior work rather than using a standardized, validated instrument. While this approach ensured alignment with the specific aims of our study and comparability with earlier research on conversational agents, it may limit the generalizability of our findings and complicate direct comparison with studies that apply standardized scales, such as the technology acceptance model. Future work should consider validating these adapted items or adopting established measures to strengthen comparability and robustness.

More work will also be needed to adapt VAs, especially speech-to-text engines, to older adults’ speech dynamics. Furthermore, speech-to-text and text-to-speech engines cannot efficiently work with dialects, such as those found in Swiss German. Swiss researchers have created speech corpora, which may be able to be used in future iterations of GRACE, to provide native language communication for Swiss German users [98].

### Conclusions

This work developed and evaluated a reiteration of GRACE that provided cognitive interventions to healthy older adults. Technical feasibility, technology acceptance, and working alliance were evaluated with overall positive assessments. Participants notably enjoyed the unambiguous interactions with GRACE, while the interventions offered were deemed appropriately challenging. Suggestions for future improvement include expanding the use of physical features, such as the screen and a button, and implementing more exercises and open-conversational abilities. This work demonstrates that embodied VAs can be positively used to deliver cognitive interventions to older adults. Results from this work will inform future iterations of GRACE, where additional interventions will be identified and tested among people with early dementia.

## Appendix

Multimedia Appendix 1 GRACE interaction script.

Multimedia Appendix 2 Large language model prompts used for GRACE interaction.

Multimedia Appendix 3 Preinteraction questionnaire (in German).

Multimedia Appendix 4 Postinteraction questionnaire (in German).

Multimedia Appendix 5 Interview questions (in German).

Multimedia Appendix 6 Direct participant quotes for questions 8-10 (translated to English).

## Abbreviations

AI: artificial intelligence
CST: cognitive stimulation therapy
ITI: intention to continue interacting
LLM: large language model
PC: perceived control
PEN: perceived enjoyment
PEU: perceived ease of use
PSA: perceived session alliance
PU: perceived usefulness
VA: voice assistant

## References

[1] (2024). What is dementia?. Alzheimer's Association.

[2] (2021). Dementia. World Health Organization.

[3] (2024). Global Burden of Disease 2021: findings from the GBD 2021 study. Institute for Health Metrics and Evaluation.

[4] Wimo A, Seeher K, Cataldi R, Cyhlarova E, Dielemann JL, Frisell O, Guerchet M, Jönsson L, Malaha AK, Nichols E, Pedroza P, Prince M, Knapp M, Dua T (2023). The worldwide costs of dementia in 2019. Alzheimers Dement.

[5] (2017). Global action plan on the public health response to dementia. World Health Organization.

[6] Schneider C, Nißen M, Kowatsch T, Vinay R (2024). Impact of digital assistive technologies on the quality of life for people with dementia: a scoping review. BMJ Open.

[7] Topo P (2008). Technology studies to meet the needs of people with dementia and their caregivers. J Appl Gerontol.

[8] Bérubé C, Schachner T, Keller R, Fleisch E, V Wangenheim F, Barata F, Kowatsch T (2021). Voice-based conversational agents for the prevention and management of chronic and mental health conditions: systematic literature review. J Med Internet Res.

[9] Shade M, Rector K, Kupzyk K (2021). Voice assistant reminders and the latency of scheduled medication use in older adults with pain: descriptive feasibility study. JMIR Form Res.

[10] Lappromrattana T, Sooraksa P (2023). Quick prototyping of companion bots for elderly people. Sens Mater.

[11] Tsiourti C, Moussa MB, Quintas J, Loke B, Jochem I, Lopes JA, Konstantas D (2016). A virtual assistive companion for older adults: design implications for a real-world application. https://link.springer.com/chapter/10.1007/978-3-319-56994-9_69.

[12] Mahmood A, Cao S, Stiber M, Antony VN, Huang C-M (2025). Voice assistants for health self-management: designing for and with older adults.

[13] Cruz-Sandova D, Morales-Tellez A, Sandoval EB, Favela J (2020). A social robot as therapy facilitator in interventions to deal with dementia-related behavioral symptoms. https://ieeexplore.ieee.org/document/9484212.

[14] Morales-de-Jesús V, Gómez-Adorno H, Somodevilla-García M, Vilariño D (2021). Conversational system as assistant tool in reminiscence therapy for people with early-stage of Alzheimer's. Healthcare (Basel).

[15] Sezgin E, Militello L, Huang Y, Lin S (2020). A scoping review of patient-facing, behavioral health interventions with voice assistant technology targeting self-management and healthy lifestyle behaviors. Transl Behav Med.

[16] Bickmore T, Cassell J, van Kuppevelt JCJ, Dybkjær L, Bernsen NO (2005). Social dialongue with embodied conversational agents. Advances in Natural Multimodal Dialogue Systems. Text, Speech and Language Technology, Vol 30.

[17] Lima MR, Wairagkar M, Gupta M, Rodriguez y Baena F, Barnaghi P, Sharp DJ, Vaidyanathan R (2022). Conversational affective social robots for ageing and dementia support. IEEE Trans Cogn Dev Syst.

[18] Saragih ID, Tonapa S, Saragih I, Lee B-O (2022). Effects of cognitive stimulation therapy for people with dementia: a systematic review and meta-analysis of randomized controlled studies. Int J Nurs Stud.

[19] Vinay R, Tommila NC, Schlögl M, Klöppel S, Biller-Andorno N, Kowatsch T (2024). Demonstrating GRACE: our embodied voice assistant providing cognitive interventions.

[20] Vinay R, Tommila NC, Schlögl M, Klöppel S, Biller-Andorno N, Kowatsch T (2024). GRACE: Towards an embodied voice assistant for improving quality of life by leveraging elements of cognitive stimulation therapy.

[21] Davis FD, Bagozzi RP, Warshaw PR (1989). User acceptance of computer technology: a comparison of two theoretical models. Manag Sci.

[22] Davis FD, Davis F (1989). Perceived usefulness, perceived ease of use, and user acceptance of information technology. MIS Q.

[23] Venkatesh V (2000). Determinants of perceived ease of use: integrating control, intrinsic motivation, and emotion into the technology acceptance model. Inf Syst Res.

[24] Falkenström F, Hatcher RL, Skjulsvik T, Larsson MH, Holmqvist R (2015). Development and validation of a 6-item working alliance questionnaire for repeated administrations during psychotherapy. Psychol Assess.

[25] Ollier J, Suryapalli P, Fleisch E, von Wangenheim F, Mair JL, Salamanca-Sanabria A, Kowatsch T (2023). Can digital health researchers make a difference during the pandemic? Results of the single-arm, chatbot-led Elena+: care for COVID-19 interventional study. Front Public Health.

[26] Brill E, Vinay R, Nißen M, Joshi P, Klöppel S, Kowatsch T (2025). Toward a smartphone-based and conversational agent-delivered just-in-time adaptive holistic lifestyle intervention for older adults affected by cognitive decline: two-week proof-of-concept study. JMIR Form Res.

[27] Kowatsch T, Schachner T, Harperink S, Barata F, Dittler U, Xiao G, Stanger C, V Wangenheim F, Fleisch E, Oswald H, Möller A (2021). Conversational agents as mediating social actors in chronic disease management involving health care professionals, patients, and family members: multisite single-arm feasibility study. J Med Internet Res.

[28] Venkatesh V, Thong JYL, Xu X (2012). Consumer acceptance and use of information technology: extending the unified theory of acceptance and use of technology. MIS Q.

[29] (2020). Assessment List for Trustworthy Artificial Intelligence (ALTAI) for self-assessment. European Commission.

[30] Harrington CN, Garg R, Woodward A, Williams D (2022). "It's kind of like code-switching": Black older adults' experiences with a voice assistant for health information seeking.

[31] Martin-Hammond A, Vemireddy S, Rao K (2019). Exploring older adults' beliefs about the use of intelligent assistants for consumer health information management: a participatory design study. JMIR Aging.

[32] Blocker KA, Kadylak T, Koon LM, Kovac CE, Rogers WA (2021). Digital home assistants and aging: initial perspectives from novice older adult users. Proc Hum Factors Ergon Soc Annu Meet.

[33] Koon LM, McGlynn SA, Blocker KA, Rogers WA (2019). Perceptions of digital assistants from early adopters aged 55+. Ergon Des.

[34] Yan Z, Dube V, Heselton J, Johnson K, Yan C, Jones V, Blaskewicz Boron J, Shade M (2024). Understanding older people's voice interactions with smart voice assistants: a new modified rule-based natural language processing model with human input. Front Digit Health.

[35] Mitzner TL, Boron JB, Fausset CB, Adams AE, Charness N, Czaja SJ, Dijkstra K, Fisk AD, Rogers WA, Sharit J (2010). Older adults talk technology: technology usage and attitudes. Comput Human Behav.

[36] Razavi SZ, Schubert LK, van Orden K, Ali MR, Kane B, Hoque E (2022). Discourse behavior of older adults interacting with a dialogue agent competent in multiple topics. ACM Trans Interact Intell Syst.

[37] Wolters M, Georgila K, Moore JD, MacPherson SE (2009). Being old doesn’t mean acting old: how older users interact with spoken dialog systems. ACM Trans Access Comput.

[38] Schlögl S, Chollet G, Garschall M, Tscheligi M, Legouverneur G (2013). Exploring voice user interfaces for seniors.

[39] Nallam P, Bhandari S, Sanders J, Martin-Hammond A (2020). A question of access: exploring the perceived benefits and barriers of intelligent voice assistants for improving access to consumer health resources among low-income older adults. Gerontol Geriatr Med.

[40] Joshi KR, Ulabhaje AA, Nataraj R, Martin-Hammond A (2025). Skeptical yet curious: attitudes towards conversational assistants for supporting social engagement among older adults living alone.

[41] Huang Y, Zhou Q, Piper AM (2025). Designing conversational AI for aging: a systematic review of older adults' perceptions and needs.

[42] Moyle W (2019). The promise of technology in the future of dementia care. Nat Rev Neurol.

[43] Marziali RA, Franceschetti C, Dinculescu A, Nistorescu A, Kristály DM, Moșoi AA, Broekx R, Marin M, Vizitiu C, Moraru S, Rossi L, Di Rosa M (2024). Reducing loneliness and social isolation of older adults through voice assistants: literature review and bibliometric analysis. J Med Internet Res.

[44] Chen Y, Zeng J, Liu Y, Zhang J, Wu X, Li H, Ying F, Jain LC, Wan R, Wu Q, Shi F (2024). Digital radio: a one-touch interactive system for elderly companionship. Frontiers in Artificial Intelligence and Applications.

[45] Rincon JA, Marco-Detchart C, Julian V, Carrascosa C, Novais P (2022). Towards a low-cost companion robot for helping elderly well-being.

[46] Miehle J, Bagci I, Minker W, Ultes S, Eskenazi M, Devillers L, Mariani J (2019). A social companion and conversational partner for the elderly. Advanced Social Interaction with Agents. Lecture Notes in Electrical Engineering, Vol 510.

[47] Sung H, Chang S, Chin M, Lee W (2015). Robot-assisted therapy for improving social interactions and activity participation among institutionalized older adults: a pilot study. Asia Pac Psychiatry.

[48] Striegl J, Gotthardt M, Loitsch C, Weber G (2022). Investigating the usability of voice assistant-based CBT for age-related depression.

[49] Wiratunga N, Cooper K, Wijekoon A, Palihawadana C, Mendham V, Reiter E, Martin K FitChat: conversational artificial intelligence interventions for encouraging physical activity in older adults. ArXiv.

[50] Favela J, Cruz-Sandoval D, Parra MO (2023). Conversational agents for dementia using large language models. https://ieeexplore.ieee.org/document/10508610/.

[51] Utami D, Bickmore T, Nikolopoulou A, Paasche-Orlow M (2017). Talk about death: end of life planning with a virtual agent.

[52] Leusmann J, Wang C, Wang G Comparing Rule-Based and LLM-Based Methods to Enable Active Robot Assistant Conversations.

[53] Porcheron M, Fischer JE, Reeves S, Sharples S (2018). Voice interfaces in everyday life.

[54] Myers C, Furqan A, Nebolsky J, Caro K, Zhu J (2018). Patterns for how users overcome obstacles in voice user interfaces.

[55] Kadam AV (2023). Using knowledge graphs and LLMs to enhance natural language understanding on voice assistants. Int J Comput Appl.

[56] Sheng Y, Zheng L, Yuan B, Li Z, Ryabinin M, Chen B, Liang P, Ré C, Stoica I, Zhang C (2023). FlexGen: high-throughput generative inference of large language models with a single GPU. https://dl.acm.org/doi/abs/10.5555/3618408.3619696.

[57] Zhang Y, Li Y, Cui L, Cai D, Liu L, Fu T, Huang X, Zhao E, Zhang Y, Xu C, Chen Y, Wang L, Luu AT, Bi W, Shi F, Shi S Siren's song in the AI ocean: a survey on hallucination in large language models. ArXiv.

[58] Bender EM, Gebru T, McMillan-Major A, Shmitchell S (2021). On the dangers of stochastic parrots: can language models be too big?.

[59] Hastings J (2024). Preventing harm from non-conscious bias in medical generative AI. Lancet Digit Health.

[60] An K, Chen Q, Deng C, Du Z, Gao C, Gao Z, Gu Y, He T, Hu H, Hu K, Ji S, Li Y, Li Z, Lu H, Luo H, Lv X, Ma B, Ma Z, Ni C, Song C, Shi J, Shi X, Wang H, Wang W, Wang Y, Xiao Z, Yan Z, Yang Y, Zhang B, Zhang Q, Zhang S, Zhao N, Zheng S FunAudioLLM: voice understanding and generation foundation models for natural interaction between humans and LLMs. ArXiv.

[61] Schalkwyk J, Kumar A, Lyth D SesameAILabs: a conversational speech generation model. GitHub.

[62] Mahmood A, Wang J, Yao B, Wang D, Huang C-M (2025). User interaction patterns and breakdowns in conversing with LLM-powered voice assistants. Int J Hum Comput Stud.

[63] Kim CY, Lee CP, Mutlu B (2024). Understanding large-language model (LLM)-powered human-robot interaction. https://dl.acm.org/doi/10.1145/3610977.3634966.

[64] Lima MR, O'Connell A, Zhou F, Nagahara, A, Hulyalkar A, Deshpande A, Thomason J, Vaidyanathan R, Matarić M (2025). Promoting cognitive health in elder care with large language model-powered socially assistive robots.

[65] Padmanabha A, Yuan J, Gupta J, Karachiwalla Z, Majidi C, Admoni H, Erickson Z (2024). VoicePilot: harnessing LLMs as speech interfaces for physically assistive robots.

[66] Yang Z, Xu X, Yao B, Rogers E, Zhang S, Intille S, Shara N, Gao GG, Wang D (2024). Talk2Care: an LLM-based voice assistant for communication between healthcare providers and older adults. Proc ACM Interact Mob Wearable Ubiquitous Technol.

[67] Marques da Rocha M, Cruz-Sandoval D, Favela J, Muchaluat-Saade DC (2024). Design and usability evaluation of the EvaSIM simulator for a socially assistive robot. Multimed Tools Appl.

[68] (2024). OpenAI Platform.

[69] (2024). EVA Social Robot. Github.

[70] (2024). ElevenLabs: AI Voice Generator.

[71] Spector A, Orrell M, Davies S, Woods B (2010). Can reality orientation be rehabilitated? Development and piloting of an evidence-based programme of cognition-based therapies for people with dementia. Neuropsychol Rehabil.

[72] Spector A, Thorgrimsen L, Woods B, Royan L, Davies S, Butterworth M, Orrell M (2003). Efficacy of an evidence-based cognitive stimulation therapy programme for people with dementia: randomised controlled trial. Br J Psychiatry.

[73] Yates L, Orrell M, Leung P, Spector A, Woods B, Orgeta V (2014). Making a Difference 3: Individualised CST? A Manual for Carers.

[74] Ryan RM, Deci EL (2000). Self-determination theory and the facilitation of intrinsic motivation, social development, and well-being. Am Psychol.

[75] Balban MY, Neri E, Kogon MM, Weed L, Nouriani B, Jo B, Holl G, Zeitzer JM, Spiegel D, Huberman AD (2023). Brief structured respiration practices enhance mood and reduce physiological arousal. Cell Rep Med.

[76] van der Heijden H (2004). User acceptance of hedonic information systems. MIS Q.

[77] Del Re AC, Flückiger C, Horvath AO, Wampold BE (2021). Examining therapist effects in the alliance-outcome relationship: a multilevel meta-analysis. J Consult Clin Psychol.

[78] Flückiger C, Del Re AC, Wampold BE, Symonds D, Horvath AO (2012). How central is the alliance in psychotherapy? A multilevel longitudinal meta-analysis. J Couns Psychol.

[79] Flückiger C, Del Re AC, Wampold BE, Horvath AO (2018). The alliance in adult psychotherapy: a meta-analytic synthesis. Psychotherapy (Chic).

[80] Horvath AO, Greenberg LS (1989). Development and validation of the Working Alliance Inventory. J Couns Psychol.

[81] Cronbach LJ (2025). Coefficient alpha and the internal structure of tests. Psychometrika.

[82] Kowatsch T, Lohse K, Erb V, Schittenhelm L, Galliker H, Lehner R, Huang EM (2021). Hybrid ubiquitous coaching with a novel combination of mobile and holographic conversational agents targeting adherence to home exercises: four design and evaluation studies. J Med Internet Res.

[83] Smith AL, Chaparro BS (2015). Smartphone text input method performance, usability, and preference with younger and older adults. Hum Factors.

[84] Kowalski J, Jaskulska A, Skorupska K, Abramczuk K, Biele C, Kopeć W, Marasek K (2019). Older adults and voice interaction: a pilot study with Google home. https://dl.acm.org/doi/10.1145/3290607.3312973.

[85] Qiu L, Saragih ID, Fick DM, Sundar SS, Abdullah S (2025). Voice assistants to deliver cognitive stimulation therapy for persons living with dementia.

[86] Manzini A, Keeling G, Alberts L, Vallor S, Morris MR, Gabriel I (2024). The code that binds us: navigating the appropriateness of human-AI assistant relationships. Proc AAAI/ACM Conf AI Ethics Soc.

[87] Chen G (2025). The role of AI driven personal assistants in geriatric care: opportunities, challenges, and future directions. Ann Gerontol Geriatr Res.

[88] Martell MJ, Baweja JA, Dreslin BD (2024). Mitigative strategies for recovering from large language model trust violations. J Cogn Eng Decis Mak.

[89] Lima MR, Horrocks S, Daniels S, Lamptey M, Harrison M, Vaidyanathan R (2023). The role of conversational AI in ageing and dementia care at home: a participatory study. https://ieeexplore.ieee.org/document/10309459.

[90] Bosco C, Otenen E, Osorio Torres J, Nguyen V, Chheda D, Peng X, Jessup NM, Himes AK, Cureton B, Lu Y, Hill CV, Hendrie HC, Barnes PA, Shih PC (2025). Designing a multimodal and culturally relevant Alzheimer disease and related dementia generative artificial intelligence tool for Black American informal caregivers: cognitive walk-through usability study. JMIR Aging.

[91] Bosco C, Shojaei F, Theisz AA, Osorio Torres J, Cureton B, Himes AK, Jessup NM, Barnes PA, Lu Y, Hendrie HC, Hill CV, Shih PC (2024). Testing 3 modalities (voice assistant, chatbot, and mobile app) to assist older African American and Black adults in seeking information on Alzheimer disease and related dementias: Wizard of Oz usability study. JMIR Form Res.

[92] Harrington CN, Egede L (2023). Trust, comfort and relatability: understanding Black older adults’ perceptions of chatbot design for health information seeking.

[93] Moharana S, Washington G, Carrington P, Harrington CN, Arai K (2024). Towards contextualizing embodiment of intelligent systems. Intelligent Systems and Applications. IntelliSys 2024. Lecture Notes in Networks and Systems, vol 1065.

[94] Douglas S, James IA, Ballard C (2018). Non-pharmacological interventions in dementia. Adv Psychiatr Treat.

[95] Phillips LJ, Reid-Arndt S, Pak Y (2010). Effects of a creative expression intervention on emotions, communication, and quality of life in persons with dementia. Nurs Res.

[96] Ma J, Wang Q, Lang Y, Lv S, Xu Y, Wei B (2023). Effectiveness of creative story therapy for dementia: a systematic review and meta-analysis. Eur J Med Res.

[97] Grimm P, Sheth J, Malhotra N (2010). Social desirability bias. Wiley International Encyclopedia of Marketing.

[98] Plüss M, Deriu J, Schraner Y, Paonessa C, Hartmann J, Schmidt L, Scheller C, Hürlimann M, Samardžic T, Vogel M, Cieliebak M (2023). A speech corpus for all Swiss German dialect regions. https://aclanthology.org/2023.acl-short.150/.

