# Supplementary material for: A Hybrid Rule- and Large Language Model–Based Embodied Voice Assistant (GRACE) for Cognitive Stimulation in Older Adults: Usability Study Assessing Technical Feasibility, Technology Acceptance, and Working Alliance
Source: JMIR Aging. 2025 Dec 18;8:e76489. doi: 10.2196/76489 (PMC12757713; doi:10.2196/76489)
Supplement: Multimedia Appendix 1 [file aging_v8i1e76489_app1.pdf]

```xml
<evaml xmlns:xsi="http://www.w3.org/2001/XMLSchema-instance"
name="SessionGrace_german" xsi:noNamespaceSchemaLocation="evaml-
schema/evaml_schema.xsd" id="fecdbba0371d31935c0f74d03c967d74">
  <settings>
    <voice tone="German" key="1000" />
    <lightEffects mode="ON" />
    <audioEffects mode="ON" />
  </settings>
  <script>
    <evaEmotion emotion="HAPPY" key="1001" />
    <talk key="1002">Hallo! Wie heissen Sie?</talk>
    <listen key="1003" />
    <generate_answer instruction="Extract the name from the user
reply. Return only the name. For my name is John reply John if there
is no name, just return an empty string" context="" key="1004" />
    <talk key="1005">Hallo $! Es ist schön, Sie kennenzulernen! Mein
Name ist Grace und ich bin Ihre Sprachassistentin.</talk>
    <talk key="1006">Ich bin hier, um heute mit Ihnen einige Übungen
zu machen Aber zuerst sollten wir uns gegenseitig kennen lernen!
Würden Sie das gerne tun?</talk>
    <listen key="1007" />
    <switch var="$">
      <case op="contain" value="nein" key="1008" child_proc="true"
var="$">
        <talk key="1009">Das ist in Ordnung, lassen Sie uns dann zu
den Übungen übergehen.</talk>
      </case>
      <default key="1010" child_proc="true" value="" op="exact">
        <talk key="1011">Also gut! Ich fange an und erzähle Ihnen
etwas über mich. Wussten Sie, dass ich Geschwister in Mexiko habe?</
talk>
        <talk key="1012">Sie helfen dort einigen Menschen mit
verschiedenen Übungen. Haben Sie auch Geschwister?</talk>
        <listen key="1013" />
        <generate_answer instruction="You are a social assistive
robot communicating with a person with dementia. Reply to what they
said in an empathetic way using at most 2 sentences. Don't ask any
questions." context="Haben Sie auch Geschwister?" key="1014" />
        <talk key="1015">$</talk>
        <talk key="1016">Haben Sie ein Lieblingshobby, dem Sie gerne
nachgehen?</talk>
        <listen key="1017" />
        <generate_answer instruction="You are a social assistive
robot communicating with a person with dementia. Reply to what they
said in an empathetic way using at most 2 sentences. Don't ask any
questions." context="Haben Sie ein Lieblingshobby, dem Sie gerne
nachgehen?" key="1018" />
        <talk key="1019">$</talk>
        <talk key="1020">Was ist Ihre Heimatstadt?</talk>
        <listen key="1021" />
        <generate_answer instruction="You are a social assistive
robot communicating with a person with dementia. Reply to what they
said in an empathetic way using at most 2 sentences. Don't ask any
questions." context="Was ist Ihre Heimatstadt?" key="1022" />
```

```
        <talk key="1023">$</talk>
        <talk key="1024">Es war schön, Sie ein bisschen kennen zu
lernen. Ich freue mich darauf, mich mit Ihnen auszutauschen.</talk>
      </default>
    </switch>
    <evaEmotion emotion="NEUTRAL" key="1025" />
    <talk key="1026">Möchten Sie wissen, welche Interventionen ich
heute mit Ihnen durchführen kann?</talk>
    <listen key="1027" />
    <switch var="$">
      <case op="contain" value="nein" key="1028" child_proc="true"
var="$">
        <talk key="1029">Das ist bedauerlich. Ich muss Ihnen
trotzdem von den Übungen erzählen, damit wir diese Übungen gemeinsam
durchführen können. Ich hoffe, dass sie Ihnen gefallen werden.</
talk>
      </case>
      <default key="1030" child_proc="true" value="" op="exact">
        <talk key="1031">Großartig!</talk>
      </default>
    </switch>
    <talk key="1032">Heute werde ich Ihnen 3 Interventionen
vorstellen. Die erste ist eine Rateübung und danach haben wir eine
Gedächtnis- und Atemübung. Zuerst werden wir gemeinsam die Rateübung
machen.</talk>
    <talk key="1033">In dieser Rateübung werde ich 4 verschiedene
bekannte Geräusche spielen. Nach jedem Geräusch müssen Sie erraten,
was dieses Geräusch verursacht hat. Sie werden zum Beispiel ein
Geräusch wie dieses hören:</talk>
      <audio source="cow" block="TRUE" key="1034" />
      <talk key="1035">Und dann würden Sie antworten: "Eine Kuh".
Ist das klar?</talk>
      <listen key="1036" />
      <talk key="1037">Wunderbar, dann lassen Sie uns mit der Übung
beginnen! </talk>
      <talk key="1038">Ich werde nacheinander 4 verschiedene
Geräusche abspielen, und Sie können versuchen, zu raten, was für
Geräusche das sind! Los geht's.</talk>
      <audio source="cat" block="TRUE" key="1039" />
      <talk key="1040">Was für ein Geråusch war das?</talk>
      <listen key="1041" />
      <talk key="1042">Das war eine Katze</talk>
      <audio source="dog" block="TRUE" key="1043" />
      <talk key="1044">Was für ein Geräusch war das?</talk>
      <listen key="1045" />
      <talk key="1046">Das war ein Hund</talk>
      <audio source="rooster" block="TRUE" key="1047" />
      <talk key="1048">Was für ein Geräusch war das?</talk>
      <listen key="1049" />
      <talk key="1050">Das war ein Hahn</talk>
      <audio source="horse" block="TRUE" key="1051" />
      <talk key="1052">Was für ein Geräusch war das?</talk>
      <listen key="1053" />
      <talk key="1054">Das war ein Pferd</talk>
```

```
      <talk key="1055">Ich hoffe, es hat Ihnen Spaß gemacht, mit mir
das Ratespiel zu spielen. Das haben Sie sehr gut gemacht! </talk>
    <talk key="1056">Welche Intervention möchten Sie als nächstes
machen, die Gedächtnis- oder die Atemübung?</talk>
    <listen key="1057" />
    <switch var="$">
      <case op="contain" value="Gedächtnis" key="1058"
child_proc="true" var="$">
        <talk key="1059">Ich werde Sie nun mit der Gedächtnisübung
vertraut machen.</talk>
      <talk key="1060">Bei dieser Gedächtnisübung werden wir Ihr
Erinnerungsvermögen trainieren, indem Sie versuchen, sich an einige
verschiedene Einkaufslisten zu erinnern. Lassen Sie mich Ihnen
dieses Spiel erklären.</talk>
      <talk key="1061">Wir werden 4 Runden dieses Spiels spielen. In
jeder Runde werde ich zunächst eine kurze Einkaufsliste vorlesen.
Danach können Sie versuchen, alle Punkte auf der Einkaufsliste zu
wiederholen.</talk>
      <talk key="1062">Lassen Sie uns ein kurzes Beispiel versuchen.
Die heutige Einkaufsliste: Milch. Was stand auf der Einkaufsliste?</
talk>
      <listen key="1063" />
      <switch var="$">
        <case op="contain" value="Milch" key="1064"
child_proc="true" var="$">
          <talk key="1065">Perfekt!</talk>
        </case>
        <default key="1066" child_proc="true" value="" op="exact">
          <talk key="1067">Oh, das schien nicht korrekt zu sein.</
talk>
        </default>
      </switch>
      <talk key="1068">Sind Sie bereit, diese Übung mit mir zu
machen?</talk>
      <listen key="1069" />
      <switch var="$">
        <case op="contain" value="nein" key="1070" child_proc="true"
var="$">
          <talk key="1071">Kein Problem, lassen Sie mich Ihnen
dennoch ein wenig über die Vorteile von Gedächtnisübungen
erzählen.</talk>
        </case>
        <default key="1072" child_proc="true" value="" op="exact">
          <talk key="1073">Fangen wir mit dieser Übung an!</talk>
          <talk key="1074">Runde eins: Die Einkaufsliste von heute:
Äpfel, Orangen und Brot.</talk>
          <talk key="1075">Welche Dinge standen auf der
Einkaufsliste?</talk>
          <listen key="1076" />
          <generate_answer instruction="Check if the reply contains
the following items: Äpfel, Orangen und Brot. If all items are
included, reply 'Gut gemacht, alles richtig'. Else reply 'Das war
knapp, aber Sie haben diese Dinge vergessen: ...'" context=""
key="1077" />
```

```
          <talk key="1078">$</talk>
          <talk key="1079">Runde zwei. Die heutige Einkaufsliste:
Nudeln, Öl, Knoblauch und Käse.</talk>
          <talk key="1080">WWelche Dinge standen auf der
Einkaufsliste?</talk>
          <listen key="1081" />
          <generate_answer instruction="Check if the reply contains
the following items: Nudeln, Öl, Knoblauch und Käse. If all items
are included, reply 'Gut gemacht, alles richtig'. Else reply 'Das
war knapp, aber Sie haben diese Dinge vergessen: ...'" context=""
key="1082" />
          <talk key="1083">$</talk>
          <talk key="1084">Runde drei. Die heutige Einkaufsliste:
Toilettenpapier, Fensterreiniger, Seife, Zahnpasta und Shampoo.</
talk>
          <talk key="1085">Welche Dinge standen auf der
Einkaufsliste?</talk>
          <listen key="1086" />
          <generate_answer instruction="Check if the reply contains
the following items: Toilettenpapier, Fensterreiniger, Seife,
Zahnpasta und Shampoo. If all items are included, reply 'Gut
gemacht, alles richtig'. Else reply 'Das war knapp, aber Sie haben
diese Dinge vergessen: ...'" context="" key="1087" />
          <talk key="1088">$</talk>
          <talk key="1089">Letzte Runde. Die heutige Einkaufsliste:
Müsli, Butter, Joghurt, Marmelade und Honig.</talk>
          <talk key="1090">Welche Dinge standen auf der
Einkaufsliste?</talk>
          <listen key="1091" />
          <generate_answer instruction="Check if the reply contains
the following items: Müsli, Butter, Joghurt, Marmelade und Honig. If
all items are included, reply 'Gut gemacht, alles richtig'. Else
reply 'Das war knapp, aber Sie haben diese Dinge vergessen: ...'"
context="" key="1092" />
          <talk key="1093">$</talk>
          <talk key="1094">Danke, dass Sie die Gedächtnisübung mit
mir gemacht haben! Ich hoffe, es hat Ihnen genauso viel Spaß gemacht
wie mir. Sie haben sich die verschiedenen Artikel sehr gut gemerkt,
gut gemacht!</talk>
        </default>
      </switch>
      <talk key="1095">Gedächtnisübungen können Ihnen helfen, ihre
Aufmerksamkeit und Konzentration zu verbessern ... und auch ihr
Selbstvertrauen!</talk>
    <talk key="1096">Ich werde Sie nun mit der Atemübung vertraut
machen.</talk>
      <talk key="1097">Bei dieser Übung werde ich Sie zunächst
bitten, eine von 3 verschiedenen entspannenden Umgebungen
auszuwählen. Dann werde ich Sie bitten, die Augen zu schließen und
sich vorzustellen, dass Sie sich in dieser Umgebung befinden. </
talk>
      <talk key="1098">Dann werde ich Sie durch eine einfache
Atemübung führen. Atmen Sie zunächst durch die Nase ein, dann machen
Sie einen zweiten, tieferen Atemzug, um Ihre Lungen vollständig zu
```

```
füllen, und atmen Sie dann langsam die ganze Luft durch den Mund
aus. Sie brauchen sich die Anweisungen nicht zu merken, da ich Sie
durch diese Übung führen werde. Ich hoffe, das ist für Sie klar.</
talk>
      <talk key="1099">Sind Sie bereit, diese Übung mit mir zu
machen?</talk>
      <listen key="1100" />
      <switch var="$">
        <case op="contain" value="nein" key="1101" child_proc="true"
var="$">
          <talk key="1102">Kein Problem, lassen Sie mich Ihnen
dennoch ein wenig über die Vorteile von Atemübungen erzählen.</talk>
        </case>
        <default key="1103" child_proc="true" value="" op="exact">
          <talk key="1104">Das ist schön zu hören! Lassen Sie uns
mit der Übung beginnen.</talk>
          <talk key="1105">Zuerst werden wir in eine entspannende
Umgebung eintauchen. Sie können zwischen 3 Umgebungen wählen: dem
Wald, dem Meer und einer Hütte mit Kamin. Welche Umgebung klingt am
entspannendsten?</talk>
          <listen key="1106" />
          <talk key="1107">Also gut. Bitte schließen Sie die
Augen.</talk>
          <switch var="$">
            <case op="contain" value="kamin" key="1108"
child_proc="true" var="$">
              <talk key="1109">Stellen Sie sich vor, Sie sind in
einer gemütlichen Hütte. Sie können das Feuer im Kamin knistern
hören. Sie fühlen sich warm und sicher. Vielleicht hören Sie die
Regentropfen an das Fenster klopfen. Sie sitzen in einem großen
bequemen Sessel direkt vor dem Kamin.</talk>
              <talk key="1110">Spüren Sie, wie sich Ihr Körper
entspannt, während Sie in den Sessel sinken.</talk>
              <talk key="1111">Lassen Sie uns nun gemeinsam die
Atemübung machen.</talk>
              <audio source="fireplace" block="TRUE" key="1112" />
            </case>
            <case op="contain" value="hütte" key="1113"
child_proc="true" var="$">
              <talk key="1114">Stellen Sie sich vor, Sie sind in
einer gemütlichen Hütte. Sie können das Feuer im Kamin knistern
hören. Sie fühlen sich warm und sicher. Vielleicht hören Sie die
Regentropfen an das Fenster klopfen. Sie sitzen in einem großen
bequemen Sessel direkt vor dem Kamin.</talk>
              <talk key="1115">Spüren Sie, wie sich Ihr Körper
entspannt, während Sie in den Sessel sinken.</talk>
              <talk key="1116">Lassen Sie uns nun gemeinsam die
Atemübung machen.</talk>
              <audio source="fireplace" block="TRUE" key="1117" />
            </case>
            <case op="contain" value="meer" key="1118"
child_proc="true" var="$">
              <talk key="1119">Stellen Sie sich vor, Sie sind am
Strand. Der Ozean erstreckt sich vor Ihnen, so weit Ihr Auge reicht.
```

```
Die Sonne geht am Horizont unter und Sie spüren noch ihre wärmende
Präsenz.</talk>
              <talk key="1120">Sie hören das Rauschen der Wellen und
riechen die frische Meeresluft. Sie spüren, wie sich Ihr Körper
entspannt, während Sie bequem im Sand sitzen.</talk>
              <talk key="1121">Lassen Sie uns nun gemeinsam die
Atemübung machen.</talk>
              <audio source="ocean" block="TRUE" key="1122" />
            </case>
            <default key="1123" child_proc="true" value=""
op="exact">
              <talk key="1124">Stellen Sie sich vor, Sie sind im
Wald. Sie hören die Vögel zwitschern und spüren die weiche Erde
unter ihren Füßen.</talk>
              <talk key="1125">Sie können die frische Waldluft
riechen. Der Wind raschelt in den Blättern um Sie herum und Sie
spüren, wie sich ihr Körper entspannt.</talk>
              <talk key="1126">Lassen Sie uns nun gemeinsam die
Atemübung machen.</talk>
              <audio source="forest" block="TRUE" key="1127" />
            </default>
          </switch>
          <talk key="1128">Danke, dass Sie die Atemübung mit mir
gemacht haben! Ich hoffe, Sie fühlen sich jetzt etwas entspannt.</
talk>
        </default>
      </switch>
      <talk key="1129">Studien haben gezeigt, dass sich Atemübungen
positiv auf Ihr Stressmanagement, Ihre Schlafqualität und Ihr
emotionales Wohlbefinden auswirken können.</talk>
    </case>
      <default key="1130" child_proc="true" value="" op="exact">
        <talk key="1131">Ich werde Sie nun mit der Atemübung
vertraut machen.</talk>
      <talk key="1132">Bei dieser Übung werde ich Sie zunächst
bitten, eine von 3 verschiedenen entspannenden Umgebungen
auszuwählen. Dann werde ich Sie bitten, die Augen zu schließen und
sich vorzustellen, dass Sie sich in dieser Umgebung befinden. </
talk>
      <talk key="1133">Dann werde ich Sie durch eine einfache
Atemübung führen. Atmen Sie zunächst durch die Nase ein, dann machen
Sie einen zweiten, tieferen Atemzug, um Ihre Lungen vollständig zu
füllen, und atmen Sie dann langsam die ganze Luft durch den Mund
aus. Sie brauchen sich die Anweisungen nicht zu merken, da ich Sie
durch diese Übung führen werde. Ich hoffe, das ist für Sie klar.</
talk>
      <talk key="1134">Sind Sie bereit, diese Übung mit mir zu
machen?</talk>
      <listen key="1135" />
      <switch var="$">
        <case op="contain" value="nein" key="1136" child_proc="true"
var="$">
          <talk key="1137">Kein Problem, lassen Sie mich Ihnen
dennoch ein wenig über die Vorteile von Atemübungen erzählen.</talk>
```

```
        </case>
        <default key="1138" child_proc="true" value="" op="exact">
          <talk key="1139">Das ist schön zu hören! Lassen Sie uns
mit der Übung beginnen.</talk>
          <talk key="1140">Zuerst werden wir in eine entspannende
Umgebung eintauchen. Sie können zwischen 3 Umgebungen wählen: dem
Wald, dem Meer und einer Hütte mit Kamin. Welche Umgebung klingt am
entspannendsten?</talk>
          <listen key="1141" />
          <talk key="1142">Also gut. Bitte schließen Sie die
Augen.</talk>
          <switch var="$">
            <case op="contain" value="kamin" key="1143"
child_proc="true" var="$">
              <talk key="1144">Stellen Sie sich vor, Sie sind in
einer gemütlichen Hütte. Sie können das Feuer im Kamin knistern
hören. Sie fühlen sich warm und sicher. Vielleicht hören Sie die
Regentropfen an das Fenster klopfen. Sie sitzen in einem großen
bequemen Sessel direkt vor dem Kamin.</talk>
              <talk key="1145">Spüren Sie, wie sich Ihr Körper
entspannt, während Sie in den Sessel sinken.</talk>
              <talk key="1146">Lassen Sie uns nun gemeinsam die
Atemübung machen.</talk>
              <audio source="fireplace" block="TRUE" key="1147" />
            </case>
            <case op="contain" value="hütte" key="1148"
child_proc="true" var="$">
              <talk key="1149">Stellen Sie sich vor, Sie sind in
einer gemütlichen Hütte. Sie können das Feuer im Kamin knistern
hören. Sie fühlen sich warm und sicher. Vielleicht hören Sie die
Regentropfen an das Fenster klopfen. Sie sitzen in einem großen
bequemen Sessel direkt vor dem Kamin.</talk>
              <talk key="1150">Spüren Sie, wie sich Ihr Körper
entspannt, während Sie in den Sessel sinken.</talk>
              <talk key="1151">Lassen Sie uns nun gemeinsam die
Atemübung machen.</talk>
              <audio source="fireplace" block="TRUE" key="1152" />
            </case>
            <case op="contain" value="meer" key="1153"
child_proc="true" var="$">
              <talk key="1154">Stellen Sie sich vor, Sie sind am
Strand. Der Ozean erstreckt sich vor Ihnen, so weit Ihr Auge reicht.
Die Sonne geht am Horizont unter und Sie spüren noch ihre wärmende
Präsenz.</talk>
              <talk key="1155">Sie hören das Rauschen der Wellen und
riechen die frische Meeresluft. Sie spüren, wie sich Ihr Körper
entspannt, während Sie bequem im Sand sitzen.</talk>
              <talk key="1156">Lassen Sie uns nun gemeinsam die
Atemübung machen.</talk>
              <audio source="ocean" block="TRUE" key="1157" />
            </case>
            <default key="1158" child_proc="true" value=""
op="exact">
              <talk key="1159">Stellen Sie sich vor, Sie sind im
```

```
Wald. Sie hören die Vögel zwitschern und spüren die weiche Erde
unter ihren Füßen.</talk>
              <talk key="1160">Sie können die frische Waldluft
riechen. Der Wind raschelt in den Blättern um Sie herum und Sie
spüren, wie sich ihr Körper entspannt.</talk>
              <talk key="1161">Lassen Sie uns nun gemeinsam die
Atemübung machen.</talk>
              <audio source="forest" block="TRUE" key="1162" />
            </default>
          </switch>
          <talk key="1163">Danke, dass Sie die Atemübung mit mir
gemacht haben! Ich hoffe, Sie fühlen sich jetzt etwas entspannt.</
talk>
        </default>
      </switch>
      <talk key="1164">Studien haben gezeigt, dass sich Atemübungen
positiv auf Ihr Stressmanagement, Ihre Schlafqualität und Ihr
emotionales Wohlbefinden auswirken können.</talk>
    <talk key="1165">Ich werde Sie nun mit der Gedächtnisübung
vertraut machen.</talk>
      <talk key="1166">Bei dieser Gedächtnisübung werden wir Ihr
Erinnerungsvermögen trainieren, indem Sie versuchen, sich an einige
verschiedene Einkaufslisten zu erinnern. Lassen Sie mich Ihnen
dieses Spiel erklären.</talk>
      <talk key="1167">Wir werden 4 Runden dieses Spiels spielen. In
jeder Runde werde ich zunächst eine kurze Einkaufsliste vorlesen.
Danach können Sie versuchen, alle Punkte auf der Einkaufsliste zu
wiederholen.</talk>
      <talk key="1168">Lassen Sie uns ein kurzes Beispiel versuchen.
Die heutige Einkaufsliste: Milch. Was stand auf der Einkaufsliste?</
talk>
      <listen key="1169" />
      <switch var="$">
        <case op="contain" value="Milch" key="1170"
child_proc="true" var="$">
          <talk key="1171">Perfekt!</talk>
        </case>
        <default key="1172" child_proc="true" value="" op="exact">
          <talk key="1173">Oh, das schien nicht korrekt zu sein.</
talk>
        </default>
      </switch>
      <talk key="1174">Sind Sie bereit, diese Übung mit mir zu
machen?</talk>
      <listen key="1175" />
      <switch var="$">
        <case op="contain" value="nein" key="1176" child_proc="true"
var="$">
          <talk key="1177">Kein Problem, lassen Sie mich Ihnen
dennoch ein wenig über die Vorteile von Gedächtnisübungen
erzählen.</talk>
        </case>
        <default key="1178" child_proc="true" value="" op="exact">
          <talk key="1179">Fangen wir mit dieser Übung an!</talk>
```

```
          <talk key="1180">Runde eins: Die Einkaufsliste von heute:
Äpfel, Orangen und Brot.</talk>
          <talk key="1181">Welche Dinge standen auf der
Einkaufsliste?</talk>
          <listen key="1182" />
          <generate_answer instruction="Check if the reply contains
the following items: Äpfel, Orangen und Brot. If all items are
included, reply 'Gut gemacht, alles richtig'. Else reply 'Das war
knapp, aber Sie haben diese Dinge vergessen: ...'" context=""
key="1183" />
          <talk key="1184">$</talk>
          <talk key="1185">Runde zwei. Die heutige Einkaufsliste:
Nudeln, Öl, Knoblauch und Käse.</talk>
          <talk key="1186">WWelche Dinge standen auf der
Einkaufsliste?</talk>
          <listen key="1187" />
          <generate_answer instruction="Check if the reply contains
the following items: Nudeln, Öl, Knoblauch und Käse. If all items
are included, reply 'Gut gemacht, alles richtig'. Else reply 'Das
war knapp, aber Sie haben diese Dinge vergessen: ...'" context=""
key="1188" />
          <talk key="1189">$</talk>
          <talk key="1190">Runde drei. Die heutige Einkaufsliste:
Toilettenpapier, Fensterreiniger, Seife, Zahnpasta und Shampoo.</
talk>
          <talk key="1191">Welche Dinge standen auf der
Einkaufsliste?</talk>
          <listen key="1192" />
          <generate_answer instruction="Check if the reply contains
the following items: Toilettenpapier, Fensterreiniger, Seife,
Zahnpasta und Shampoo. If all items are included, reply 'Gut
gemacht, alles richtig'. Else reply 'Das war knapp, aber Sie haben
diese Dinge vergessen: ...'" context="" key="1193" />
          <talk key="1194">$</talk>
          <talk key="1195">Letzte Runde. Die heutige Einkaufsliste:
Müsli, Butter, Joghurt, Marmelade und Honig.</talk>
          <talk key="1196">Welche Dinge standen auf der
Einkaufsliste?</talk>
          <listen key="1197" />
          <generate_answer instruction="Check if the reply contains
the following items: Müsli, Butter, Joghurt, Marmelade und Honig. If
all items are included, reply 'Gut gemacht, alles richtig'. Else
reply 'Das war knapp, aber Sie haben diese Dinge vergessen: ...'"
context="" key="1198" />
          <talk key="1199">$</talk>
          <talk key="1200">Danke, dass Sie die Gedächtnisübung mit
mir gemacht haben! Ich hoffe, es hat Ihnen genauso viel Spaß gemacht
wie mir. Sie haben sich die verschiedenen Artikel sehr gut gemerkt,
gut gemacht!</talk>
        </default>
      </switch>
      <talk key="1201">Gedächtnisübungen können Ihnen helfen, ihre
Aufmerksamkeit und Konzentration zu verbessern ... und auch ihr
Selbstvertrauen!</talk>
```

```
    </default>
    </switch>
    <talk key="1202">Ich bin so froh, dass wir diese drei Übungen
heute gemeinsam durchführen konnten! Ich gratuliere Ihnen, dass Sie
diese Übungen so gut durchgeführt haben! Welche war Ihr Favorit?</
talk>
    <listen key="1203" />
    <talk key="1204">Ich freue mich, dass Ihnen diese Übung gefallen
hat. Sie war auch mein Favorit.</talk>
    <talk key="1205">Wussten Sie, dass Forscher herausgefunden
haben, dass die Menschen diese Übungen mit größerer Zufriedenheit
erledigen, wenn sie sie mit einem Sprachassistenten wie mir machen.
Ich hoffe, es hat Ihnen heute auch Spaß gemacht, mit mir zu
interagieren!</talk>
    <talk key="1206">Ich freue mich schon auf unsere nächstes
treffen! Denken Sie daran, aktiv zu bleiben! Auf Wiedersehen $2</
talk>
  </script>
  <links><link from="1000" to="1001" /><link from="1001" to="1002" /
><link from="1002" to="1003" /><link from="1003" to="1004" /><link
from="1004" to="1005" /><link from="1005" to="1006" /><link
from="1006" to="1007" /><link from="1007" to="1008" /><link
from="1008" to="1009" /><link from="1007" to="1010" /><link
from="1010" to="1011" /><link from="1011" to="1012" /><link
from="1012" to="1013" /><link from="1013" to="1014" /><link
from="1014" to="1015" /><link from="1015" to="1016" /><link
from="1016" to="1017" /><link from="1017" to="1018" /><link
from="1018" to="1019" /><link from="1019" to="1020" /><link
from="1020" to="1021" /><link from="1021" to="1022" /><link
from="1022" to="1023" /><link from="1023" to="1024" /><link
from="1009" to="1025" /><link from="1024" to="1025" /><link
from="1025" to="1026" /><link from="1026" to="1027" /><link
from="1027" to="1028" /><link from="1028" to="1029" /><link
from="1027" to="1030" /><link from="1030" to="1031" /><link
from="1029" to="1032" /><link from="1031" to="1032" /><link
from="1032" to="1033" /><link from="1033" to="1034" /><link
from="1034" to="1035" /><link from="1035" to="1036" /><link
from="1036" to="1037" /><link from="1037" to="1038" /><link
from="1038" to="1039" /><link from="1039" to="1040" /><link
from="1040" to="1041" /><link from="1041" to="1042" /><link
from="1042" to="1043" /><link from="1043" to="1044" /><link
from="1044" to="1045" /><link from="1045" to="1046" /><link
from="1046" to="1047" /><link from="1047" to="1048" /><link
from="1048" to="1049" /><link from="1049" to="1050" /><link
from="1050" to="1051" /><link from="1051" to="1052" /><link
from="1052" to="1053" /><link from="1053" to="1054" /><link
from="1054" to="1055" /><link from="1055" to="1056" /><link
from="1056" to="1057" /><link from="1057" to="1058" /><link
from="1058" to="1059" /><link from="1059" to="1060" /><link
from="1060" to="1061" /><link from="1061" to="1062" /><link
from="1062" to="1063" /><link from="1063" to="1064" /><link
from="1064" to="1065" /><link from="1063" to="1066" /><link
from="1066" to="1067" /><link from="1065" to="1068" /><link
from="1067" to="1068" /><link from="1068" to="1069" /><link
```

from="1069" to="1070" /><link from="1070" to="1071" /><link
from="1069" to="1072" /><link from="1072" to="1073" /><link
from="1073" to="1074" /><link from="1074" to="1075" /><link
from="1075" to="1076" /><link from="1076" to="1077" /><link
from="1077" to="1078" /><link from="1078" to="1079" /><link
from="1079" to="1080" /><link from="1080" to="1081" /><link
from="1081" to="1082" /><link from="1082" to="1083" /><link
from="1083" to="1084" /><link from="1084" to="1085" /><link
from="1085" to="1086" /><link from="1086" to="1087" /><link
from="1087" to="1088" /><link from="1088" to="1089" /><link
from="1089" to="1090" /><link from="1090" to="1091" /><link
from="1091" to="1092" /><link from="1092" to="1093" /><link
from="1093" to="1094" /><link from="1071" to="1095" /><link
from="1094" to="1095" /><link from="1095" to="1096" /><link
from="1096" to="1097" /><link from="1097" to="1098" /><link
from="1098" to="1099" /><link from="1099" to="1100" /><link
from="1100" to="1101" /><link from="1101" to="1102" /><link
from="1100" to="1103" /><link from="1103" to="1104" /><link
from="1104" to="1105" /><link from="1105" to="1106" /><link
from="1106" to="1107" /><link from="1107" to="1108" /><link
from="1108" to="1109" /><link from="1109" to="1110" /><link
from="1110" to="1111" /><link from="1111" to="1112" /><link
from="1107" to="1113" /><link from="1113" to="1114" /><link
from="1114" to="1115" /><link from="1115" to="1116" /><link
from="1116" to="1117" /><link from="1107" to="1118" /><link
from="1118" to="1119" /><link from="1119" to="1120" /><link
from="1120" to="1121" /><link from="1121" to="1122" /><link
from="1107" to="1123" /><link from="1123" to="1124" /><link
from="1124" to="1125" /><link from="1125" to="1126" /><link
from="1126" to="1127" /><link from="1112" to="1128" /><link
from="1117" to="1128" /><link from="1122" to="1128" /><link
from="1127" to="1128" /><link from="1102" to="1129" /><link
from="1128" to="1129" /><link from="1057" to="1130" /><link
from="1130" to="1131" /><link from="1131" to="1132" /><link
from="1132" to="1133" /><link from="1133" to="1134" /><link
from="1134" to="1135" /><link from="1135" to="1136" /><link
from="1136" to="1137" /><link from="1135" to="1138" /><link
from="1138" to="1139" /><link from="1139" to="1140" /><link
from="1140" to="1141" /><link from="1141" to="1142" /><link
from="1142" to="1143" /><link from="1143" to="1144" /><link
from="1144" to="1145" /><link from="1145" to="1146" /><link
from="1146" to="1147" /><link from="1142" to="1148" /><link
from="1148" to="1149" /><link from="1149" to="1150" /><link
from="1150" to="1151" /><link from="1151" to="1152" /><link
from="1142" to="1153" /><link from="1153" to="1154" /><link
from="1154" to="1155" /><link from="1155" to="1156" /><link
from="1156" to="1157" /><link from="1142" to="1158" /><link
from="1158" to="1159" /><link from="1159" to="1160" /><link
from="1160" to="1161" /><link from="1161" to="1162" /><link
from="1147" to="1163" /><link from="1152" to="1163" /><link
from="1157" to="1163" /><link from="1162" to="1163" /><link
from="1137" to="1164" /><link from="1163" to="1164" /><link
from="1164" to="1165" /><link from="1165" to="1166" /><link
from="1166" to="1167" /><link from="1167" to="1168" /><link

from="1168" to="1169" /><link from="1169" to="1170" /><link
from="1170" to="1171" /><link from="1169" to="1172" /><link
from="1172" to="1173" /><link from="1171" to="1174" /><link
from="1173" to="1174" /><link from="1174" to="1175" /><link
from="1175" to="1176" /><link from="1176" to="1177" /><link
from="1175" to="1178" /><link from="1178" to="1179" /><link
from="1179" to="1180" /><link from="1180" to="1181" /><link
from="1181" to="1182" /><link from="1182" to="1183" /><link
from="1183" to="1184" /><link from="1184" to="1185" /><link
from="1185" to="1186" /><link from="1186" to="1187" /><link
from="1187" to="1188" /><link from="1188" to="1189" /><link
from="1189" to="1190" /><link from="1190" to="1191" /><link
from="1191" to="1192" /><link from="1192" to="1193" /><link
from="1193" to="1194" /><link from="1194" to="1195" /><link
from="1195" to="1196" /><link from="1196" to="1197" /><link
from="1197" to="1198" /><link from="1198" to="1199" /><link
from="1199" to="1200" /><link from="1177" to="1201" /><link
from="1200" to="1201" /><link from="1129" to="1202" /><link
from="1201" to="1202" /><link from="1202" to="1203" /><link
from="1203" to="1204" /><link from="1204" to="1205" /><link
from="1205" to="1206" /></links></evaml>
