# Supplementary material for: A Hybrid Rule- and Large Language Model–Based Embodied Voice Assistant (GRACE) for Cognitive Stimulation in Older Adults: Usability Study Assessing Technical Feasibility, Technology Acceptance, and Working Alliance
Source: JMIR Aging. 2025 Dec 18;8:e76489. doi: 10.2196/76489 (PMC12757713; doi:10.2196/76489)
Supplement: Multimedia Appendix 3 [file aging_v8i1e76489_app3.pdf]

Studie: GRACE, August 2024
FRAGEBOGEN (1 von 2)

ID: ______________

Bitte lesen Sie die folgenden Fragen durch und geben Sie Ihre Antwort anhand der bereitgestellten Skalen an.

Bitte wählen Sie Ihr Geschlecht:

- ☐ Mann
- ☐ Frau
- ☐ Andere: ________________
- ☐ Möchte ich nicht sagen

Bitte geben Sie Ihr Alter ein:

____

INFORMATIONEN:

Ein Sprachassistent ist ein Gerät (oder eine Software), das auf Sprachbefehle reagiert und Ihnen bei der Ausführung kleiner Aufgaben oder der Beantwortung Ihrer Fragen helfen kann. Sie sind auf Ihren Smartphones, Computern, intelligenten Lautsprechern oder sogar auf Smartwatches zu finden. Bitte beachten Sie diese Informationen, wenn Sie die folgenden Fragen beantworten.

1. Ich habe schon einmal einen Sprachassistenten verwendet (z. B. Siri, Alexa, Google Assistant, etc.).

Ja	Nein
☐	☐

2. Wie oft verwenden Sie einen Sprachassistenten (z. B. auf Ihrem Telefon, in einem intelligenten Lautsprecher, etc.)?

Nie	Einmal im Monat	Ein paar Mal im Monat	Einmal am Tag	Ein paar Mal am Tag
☐	☐	☐	☐	☐

3. Wenn mir ein Sprachassistent Übungen und Aktivitäten anbieten könnte, die meine Lebensqualität verbessern würden, würde ich ihn nutzen.

stimme überhaupt nicht zu	stimme nicht zu	stimme eher nicht zu	stimme weder noch zu	stimme eher zu	stimme zu	stimme voll und ganz zu
☐	☐	☐	☐	☐	☐	☐

Vielen Dank für das Ausfüllen von Fragebogen 1.
