# Supplementary material for: A Hybrid Rule- and Large Language Model–Based Embodied Voice Assistant (GRACE) for Cognitive Stimulation in Older Adults: Usability Study Assessing Technical Feasibility, Technology Acceptance, and Working Alliance
Source: JMIR Aging. 2025 Dec 18;8:e76489. doi: 10.2196/76489 (PMC12757713; doi:10.2196/76489)
Supplement: Multimedia Appendix 4 [file aging_v8i1e76489_app4.pdf]

Studie: GRACE, August 2024
FRAGEBOGEN (2 von 2)

ID: ____________

Bitte lesen Sie die folgenden Fragen und geben Sie Ihre Antwort mithilfe der bereitgestellten Skalen ein.

Wie haben Sie die Interaktion mit GRACE wahrgenommen?

1. Ich fand die GRACE-Interaktion einfach.

stimme überhaupt nicht zu	stimme nicht zu	stimme eher nicht zu	stimme weder noch zu	stimme eher zu	stimme zu	stimme voll und ganz zu
☐	☐	☐	☐	☐	☐	☐

2. Die Interaktion mit GRACE hat mir Spass gemacht.

stimme überhaupt nicht zu	stimme nicht zu	stimme eher nicht zu	stimme weder noch zu	stimme eher zu	stimme zu	stimme voll und ganz zu
☐	☐	☐	☐	☐	☐	☐

3. Ich fand es nützlich, die Interventionen mit GRACE durchzuführen.

stimme überhaupt nicht zu	stimme nicht zu	stimme eher nicht zu	stimme weder noch zu	stimme eher zu	stimme zu	stimme voll und ganz zu
☐	☐	☐	☐	☐	☐	☐

4. GRACE hat mich motiviert die Interventionen durchzuführen.

stimme überhaupt nicht zu	stimme nicht zu	stimme eher nicht zu	stimme weder noch zu	stimme eher zu	stimme zu	stimme voll und ganz zu
☐	☐	☐	☐	☐	☐	☐

5. Ich konnte meine Interaktion mit GRACE kontrollieren.

stimme überhaupt nicht zu	stimme nicht zu	stimme eher nicht zu	stimme weder noch zu	stimme eher zu	stimme zu	stimme voll und ganz zu
☐	☐	☐	☐	☐	☐	☐

6. Ich würde gerne weiterhin mit GRACE interagieren.

stimme überhaupt nicht zu	stimme nicht zu	stimme eher nicht zu	stimme weder noch zu	stimme eher zu	stimme zu	stimme voll und ganz zu
☐	☐	☐	☐	☐	☐	☐

Bitte beurteilen Sie Ihre Beziehung mit GRACE?

7. GRACE und ich haben uns respektiert.

stimme überhaupt nicht zu	stimme nicht zu	stimme eher nicht zu	stimme weder noch zu	stimme eher zu	stimme zu	stimme voll und ganz zu
☐	☐	☐	☐	☐	☐	☐

8. Ich hatte das Gefühl, dass GRACE mich wertschätzt.

stimme überhaupt nicht zu	stimme nicht zu	stimme eher nicht zu	stimme weder noch zu	stimme eher zu	stimme zu	stimme voll und ganz zu
☐	☐	☐	☐	☐	☐	☐

9. Ich hatte das Gefühl, dass GRACE sich um mich kümmert, auch wenn ich Dinge tue, die GRACE nicht gut findet.

stimme überhaupt nicht zu	stimme nicht zu	stimme eher nicht zu	stimme weder noch zu	stimme eher zu	stimme zu	stimme voll und ganz zu
☐	☐	☐	☐	☐	☐	☐

10. GRACE und ich arbeiteten auf gemeinsam vereinbarte Ziele hin.

stimme überhaupt nicht zu	stimme nicht zu	stimme eher nicht zu	stimme weder noch zu	stimme eher zu	stimme zu	stimme voll und ganz zu
☐	☐	☐	☐	☐	☐	☐

11. GRACE und ich sind uns einig, woran es für mich wichtig ist zu arbeiten.

stimme überhaupt nicht zu	stimme nicht zu	stimme eher nicht zu	stimme weder noch zu	stimme eher zu	stimme zu	stimme voll und ganz zu
☐	☐	☐	☐	☐	☐	☐

12. Ich denke, dass unsere Herangehensweise mein Problem zu lösen korrekt ist.

stimme überhaupt nicht zu	stimme nicht zu	stimme eher nicht zu	stimme weder noch zu	stimme eher zu	stimme zu	stimme voll und ganz zu
☐	☐	☐	☐	☐	☐	☐

Vielen Dank für das Ausfüllen von Fragebogen 2.
