# Supplementary material for: A Hybrid Rule- and Large Language Model–Based Embodied Voice Assistant (GRACE) for Cognitive Stimulation in Older Adults: Usability Study Assessing Technical Feasibility, Technology Acceptance, and Working Alliance
Source: JMIR Aging. 2025 Dec 18;8:e76489. doi: 10.2196/76489 (PMC12757713; doi:10.2196/76489)
Supplement: Multimedia Appendix 5 [file aging_v8i1e76489_app5.pdf]

Studie: GRACE, August 2024
INTERVIEW

ID: ______________

“Die folgenden Fragen beziehen sich auf den GRACE-Prototyp und werden uns bei zukünftigen Studien hilfreich sein. Bitte geben Sie für jede Frage eine kurze Antwort.”

1. Hatten Sie das Gefühl, dass GRACE Ihre Fähigkeit unterstützt hat, Ihre eigenen Entscheidungen zu treffen? Können Sie ein Beispiel liefern?

2. Wie wohl haben Sie sich dabei gefühlt, persönliche Informationen mit GRACE zu teilen?

3. Glauben Sie, dass GRACE alle Benutzer fair behandelt, unabhängig von ihrem Hintergrund, ihren Fähigkeiten oder Vorlieben?

4. Wie zugänglich finden Sie die Features und Funktionen von GRACE? Gibt es bestimmte Features, die Sie besonders nützlich oder herausfordernd finden?

5. Hatten Sie jemals das Gefühl, dass die Interaktion mit GRACE Ihr körperliches oder psychisches Wohlbefinden in irgendeiner Weise beeinflusst?

6. Ist es für Sie immer klar, wenn Sie mit GRACE und nicht mit einem Menschen interagieren? Wie wirkt sich diese Klarheit auf Ihre Nutzung des Systems aus?

7. Glauben Sie, dass für den Betrieb und die Entscheidungsprozesse von GRACE eine ausreichende menschliche Aufsicht besteht?

8. Was fanden Sie besonders gut an der Interaktion mit GRACE?

9. Was müsste unbedingt an der Interaktion mit GRACE verbessert werden?

10. Haben Sie weitere Kommentare bzw. Verbesserungsvorschläge zum Studienablauf?

Vielen Dank für Ihre Antworten und Ihre heutige Teilnahme an unserer Studie.

ENGLISH TRANSLATIONS:

1. [HUMAN AGENCY: Decision-making and control] Did you feel that GRACE supported your ability to make your own decisions? Can you provide an example?

2. [PRIVACY AND DATA GOVERNANCE: Data rights] How comfortable did you feel sharing personal information with GRACE?

3. [FAIRNESS: Avoiding bias] Do you believe GRACE treats all users fairly, regardless of their background, abilities, or preferences?

4. [FAIRNESS: Accessibility] How accessible do you find GRACE's features and functionalities? Are there any specific features that you find particularly useful or challenging?

5. [WELL-BEING: Health and safety] Have you ever felt that interacting with GRACE affected your physical or psychological well-being in any way?

6. [TRANSPARENCY: Interaction awareness] Is it always clear to you when you are interacting with GRACE as opposed to a human? How does this clarity affect your use of the system?

7. [ACCOUNTABILITY: Oversight] Do you feel that there is adequate human oversight in GRACE's operation and decision-making processes?

8. [Positive Aspects] What did you particularly like about the interaction with GRACE?

9. [Suggestions for improvement] What needs to be improved about the interaction with GRACE?

10. [Final Comments and suggestions] Do you have any further comments or suggestions for improvement regarding the course of study?
