# Supplementary material for: A Hybrid Rule- and Large Language Model–Based Embodied Voice Assistant (GRACE) for Cognitive Stimulation in Older Adults: Usability Study Assessing Technical Feasibility, Technology Acceptance, and Working Alliance
Source: JMIR Aging. 2025 Dec 18;8:e76489. doi: 10.2196/76489 (PMC12757713; doi:10.2196/76489)
Supplement: Multimedia Appendix 6 [file aging_v8i1e76489_app6.pdf]

Direct participant quotes for questions 8-10 (translated to English)

Question	Participant quote
"What did you like about the interaction with GRACE?"	P01: "It was clear and unambiguous"<br>P01: "It was well-managed and well-explained"<br>P01: "It was easy"<br>P02: "GRACE was sympathetic"<br>P02: "It was bigger than I imagined"<br>P02: "I felt an immediate connection"<br>P03: "I liked her appearance and her voice"<br>P03: "The exercises were nice"<br>P03: "The explanations were helpful"<br>P04: "She was positive"<br>P04: "You didn't have stress interacting with it"<br>P04: "It was overall nice and polite"<br>P05: "I can imagine that it can be useful"<br>P05: "I like that you can do it at your own rhythm"<br>P05: "It's nice that it gives feedback"<br>P06: "You have the full attention of something, even if it's just a pseudo-interest"<br>P07: "Sometimes it gives you specific answers"<br>P08: "It has a pleasant voice"<br>P08: "There is enough time and it's not rushed"<br>P08: "It's quite pleasant and friendly"<br>P09: "It's a good thing that it's a game"<br>P09: "It's very interesting and exciting"<br>P10: "It's something new to learn"<br>P10: "I like that she could understand everything"<br>P11: "I liked the memory exercise"<br>P11: "She praised me even when I was wrong, that was motivating"<br>P12: "Even though it's a machine, it could be helpful to have an entity that asks questions and listens or hears you, even though it's not a human interaction"<br>P12: "It's great for those who are lonely"<br>P13: "She is directive but still open"<br>P13: "She would respond to me even if I mumbled"<br>P13: "In the ideal setting, without dementia, everything was okay"<br>P14: "GRACE understands what you say, and her answers were correct"<br>P15: "I appreciated the clear information and announcement"<br>P15: "It was stress-free"<br>P16: "GRACE's voice is pleasant"<br>P16: "She has clear pronounciation"<br>P16: "Sweet appearance"<br>P17: "I liked the winking"<br>P17: "She thanked me again and again"<br>P18: "I liked the beginning interaction, where we got to know each other personally"<br>P19: "The breathing exercise was good"<br>P19: "The memory questions were difficult, but also good"<br>P19: "She has a cute appearance"<br>P20: "I like the possibilities that lie within the system"<br>P20: "It has a lot of potential"<br>P21: "It had clear pronounciation"<br>P21: "Overall it was nice"<br>P21: "It looks so cute"
"What needs to be improved about the interaction with GRACE?"	P01: "The voice was too technical"<br>P01: "The lamp should be brighter"<br>P01: "Maybe her screen could be used"<br>P02: "The tasks and exercises were quite demanding"<br>P02: "For the breathing exercise, the position was not good. She should tell the participant to take a comfortable position"

	P03: "She should repeat everything again in the memory game"<br>P03: "More exercises"<br>P03: "Maybe it could explain the effects more, why should I do these exercises?"<br>P04: "Getting to know each other felt too superficial"<br>P04: "Too positive in the word choices"<br>P05: "The voice pitch was a bit disturbing, something not alive"<br>P06: "It could have more exercises"<br>P06: "I didn't like the breathing exercise"<br>P07: "It didn't always understand what I said"<br>P07: "Could be more motivating"<br>P07: "The tone and speech were not so pleasant"<br>P08: "It could have more flexibility and free choices"<br>P08: "The answers were very stereotypical, could be more variable and not always say 'well done' to me"<br>P09: "Her voice was not clear, could be brighter"<br>P09: "I had to wait long for an answer"<br>P10: "Nothing"<br>P11: "There was no personal connection"<br>P11: "The exercises could be personalized"<br>P11: "Maybe she could provide a massage"<br>P12: "Could have more human appearance, but not too much as it causes confusion. For instance you can have a human face since it's only a screen."<br>P12: "It would feel more comfortable when it's something human or familiar"<br>P12: "She had problems listening to me"<br>P13: "You should individualize the tempo in the breathing exercise"<br>P14: "It could understand better and be more precise"<br>P14: "I think it can be improved, there are various problems in old age"<br>P15: "Nothing, I could live with it"<br>P16: "The breathing exercise was too fast"<br>P17: "No not at the moment"<br>P18: "I'm not sure"<br>P19: "Her answers were a bit fast"<br>P20: "She could listen a bit better"<br>P20: "Her reaction is a bit slow"<br>P20: "It is still somewhat mechanical, it's not perfect communication"<br>P21: "The electronics are very sensitive"<br>P21: "The delay could be improved"<br>P21: "The communication is not great yet. For example it could also provide reminders for type-2 diabetes and the injections, measuring, reminding for medications, remembering to drink water, flushing the toilet, turning off the stove"
"Any further comments or suggestions for improvement?"	P01: "Why not just use Siri?"<br>P01: "Nothing else"<br>P02: "No"<br>P03: "No"<br>P04: "More background noise for the breathing exercise"<br>P04: "It looks very inviting"<br>P04: "The memory training with shopping list is great"<br>P05: "No"<br>P06: "Maybe GRACE could play a game"<br>P06: "The face and human aspects are good, especially for older people"<br>P06: "I find it interesting"<br>P07: "The breathing exercise was not relaxing"<br>P07: "The light is a bit difficult to see"<br>P08: "I would like a button"<br>P08: "Human face would be good or to be able to choose a photo, like of my grandson"<br>P09: "No"<br>P10: "No"

	P11: "No"<br>P12: "If it has more of a human appearance, then you need to clarify that it's not human"<br>P12: "You could open the possibility of topics to make it more flexible and not only have pre-established exercises"<br>P12: "Maybe also some more open exchanges"<br>P13: "No, it was quite efficient and direct"<br>P13: "Maybe give also a choice for a nice, male voice"<br>P14: "The interaction with GRACE was a bit short"<br>P15: "The study flyer was distributed too late"<br>P16: "No it was good"<br>P17: "No"<br>P18: "Was all okay"<br>P19: "No everything was okay, well done"<br>P20: "I can't yet imagine how GRACE would interact with people with dementia"<br>P20: "people with dementia cannot provide information"<br>P20: "Find some keywords, for example, my mother responded to 'garden', 'dog', 'favorite food', 'music' - bringing out invigorating melodies. GRACE's productivity is not just about reducing loneliness"<br>P21: "No"
